# Characterizing and Managing Missing Structured Data in Electronic Health Records: Data Analysis

**DOI:** 10.2196/medinform.8960

**Published:** 2018-02-23

**Authors:** Brett K Beaulieu-Jones, Daniel R Lavage, John W Snyder, Jason H Moore, Sarah A Pendergrass, Christopher R Bauer

**Affiliations:** ^1^ Genomics and Computational Biology Graduate Group Perelman School of Medicine University of Pennsylvania Philadelphia, PA United States; ^2^ Institute for Biomedical Informatics University of Pennsylvania Philadelphia, PA United States; ^3^ Biomedical and Translational Informatics Institute Geisinger Danville, PA United States

**Keywords:** imputation, missing data, clinical laboratory test results, electronic health records

## Abstract

**Background:**

Missing data is a challenge for all studies; however, this is especially true for electronic health record (EHR)-based analyses. Failure to appropriately consider missing data can lead to biased results. While there has been extensive theoretical work on imputation, and many sophisticated methods are now available, it remains quite challenging for researchers to implement these methods appropriately. Here, we provide detailed procedures for when and how to conduct imputation of EHR laboratory results.

**Objective:**

The objective of this study was to demonstrate how the mechanism of missingness can be assessed, evaluate the performance of a variety of imputation methods, and describe some of the most frequent problems that can be encountered.

**Methods:**

We analyzed clinical laboratory measures from 602,366 patients in the EHR of Geisinger Health System in Pennsylvania, USA. Using these data, we constructed a representative set of complete cases and assessed the performance of 12 different imputation methods for missing data that was simulated based on 4 mechanisms of missingness (missing completely at random, missing not at random, missing at random, and real data modelling).

**Results:**

Our results showed that several methods, including variations of Multivariate Imputation by Chained Equations (MICE) and softImpute, consistently imputed missing values with low error; however, only a subset of the MICE methods was suitable for multiple imputation.

**Conclusions:**

The analyses we describe provide an outline of considerations for dealing with missing EHR data, steps that researchers can perform to characterize missingness within their own data, and an evaluation of methods that can be applied to impute clinical data. While the performance of methods may vary between datasets, the process we describe can be generalized to the majority of structured data types that exist in EHRs, and all of our methods and code are publicly available.

## Introduction

### Justification

Missing data present a challenge to researchers in many fields, and this challenge is growing as datasets increase in size and scope. This is especially problematic for electronic health records (EHRs), where missing values frequently outnumber observed values. EHRs were designed to record and improve patient care and streamline billing, and not as resources for research [1]; thus, there are significant challenges to using these data to gain a better understanding of human health. As EHR data become increasingly used as a source of phenotypic information for biomedical research [2], it is crucial to develop strategies for coping with missing data.

Clinical laboratory assay results are a particularly rich data source within the EHR, but they also tend to have large amounts of missing data. These data may be missing for many different reasons. Some tests are used for routine screening, but screening may be biased. Other tests are only conducted if they are clinically relevant to very specific ailments. Patients may also receive care at multiple health care systems, resulting in information gaps at each institution. Age, sex, socioeconomic status, access to care, and medical conditions can all affect how comprehensive the data are for a given patient. Accounting for the mechanisms that cause data to be missing is critical, since failure to do so can lead to biased conclusions.

### Background

Aside from the uncertainty associated with a variable that is not observed, many analytical methods, such as regression or principal components analysis, are designed to operate only on a complete dataset. The easiest way to implement these procedures is to remove variables with missing values or remove individuals with missing values. Eliminating variables is justifiable in many situations, especially if a given variable has a large proportion of missing values, but doing so may restrict the scope and power of a study. Removing individuals with missing data is another option known as complete-case analysis. This is generally not recommended unless the fraction of individuals that will be removed is small enough to be considered trivial, or there is good reason to believe that the absence of a value is due to random chance. If there are systematic differences between individuals with and without observations, complete-case analysis will be biased.

**Figure 1:**
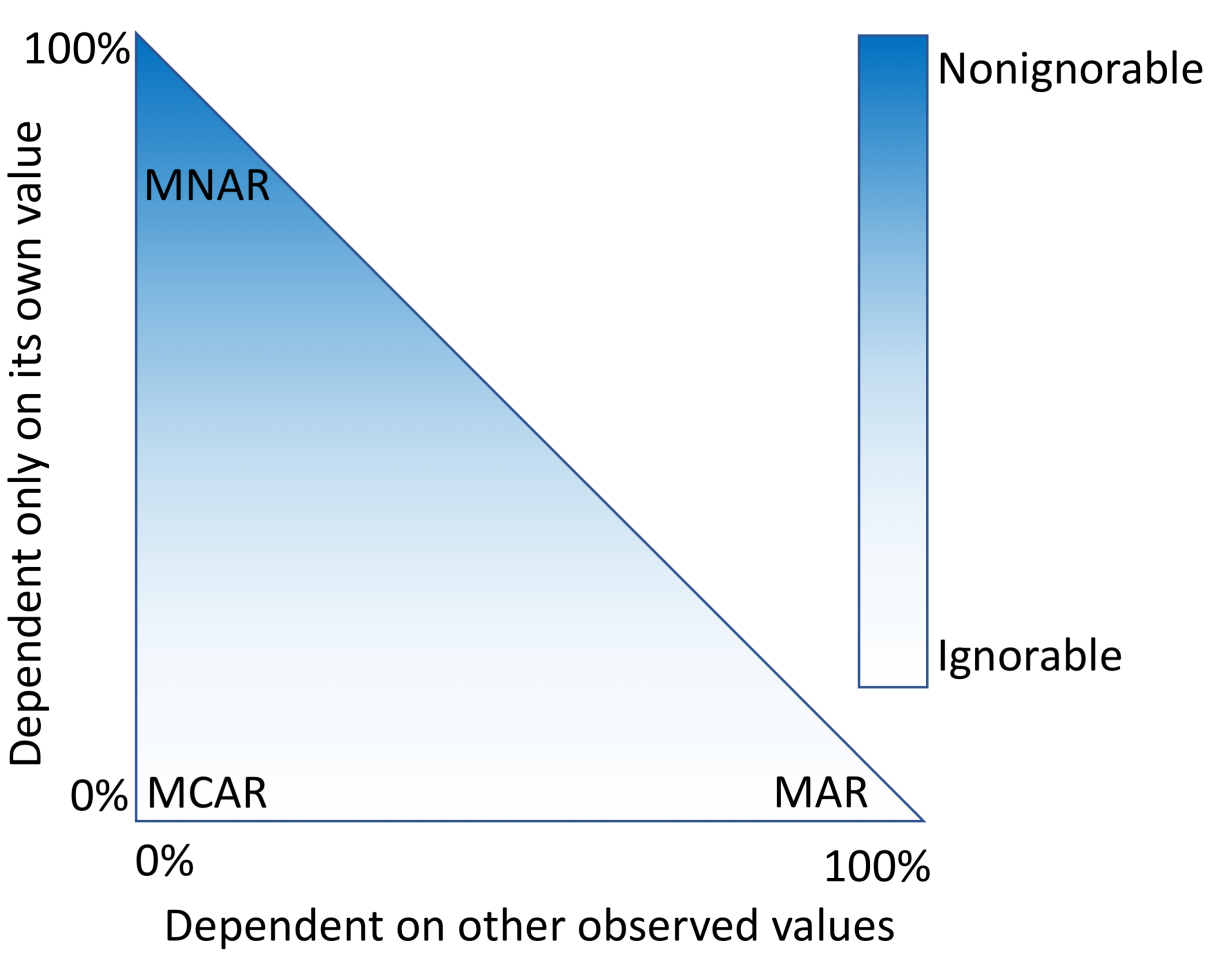
Two general paradigms are commonly used to describe missing data. Missing data are considered ignorable if the probability of observing a variable has no relation to the value of the observed variable and are considered nonignorable otherwise. The second paradigm divides missingness into 3 categories: missing completely at random (MCAR: the probability of observing a variable is not dependent on its value or other observed values), missing at random (MAR: the probability of observing a variable is not dependent on its own value after conditioning on other observed variables), and missing not at random (MNAR: the probability of observing a variable is dependent on its value, even after conditioning on other observed variables). The x-axis indicates the extent to which a given value being observed depends on other values of other observed variables. The y-axis indicates the extent to which a given value being observed depends on its own value.

An alternative approach is to fill in the fields that are missing data with estimates. This process, called imputation, requires a model that makes assumptions about why only some values were observed. Missingness mechanisms fall somewhere in a spectrum between 3 scenarios (Figure 1).

When data are missing in a manner completely unrelated to both the observed and unobserved values, they are considered to be missing completely at random (MCAR) [3,4]. When data are MCAR, the observed data represent a random sample of the population, but this is rarely encountered in practice. Conversely, data missing not at random (MNAR) refers to a situation where the probability of observing a data point depends on the value of that data point [5]. In this case, the mechanism responsible for the missing data is biased and should not be considered ignorable [6]. For example, rheumatoid factor is an antibody detectable in blood, and the concentration of this antibody is correlated with the presence and severity of rheumatoid arthritis. This test is typically performed only for patients with some indication of rheumatoid arthritis. Thus, patients with high rheumatoid factor levels are more likely to have rheumatoid factor measures.

A more complicated scenario can arise when multiple variables are available. If the probability of observing a data point does not depend on the value of that data point, after conditioning on 1 or more additional variables, then that data point is said to be missing at random (MAR) [5]. For example, a variable, *X,* may be MNAR if considered in isolation. However, if we observe another variable, *Y*, that explains some of the variation in *X* such that, after conditioning on *Y*, the probability of observing *X* is no longer related to its own value, then *X* is said to be MAR. In this way, *Y* can transform *X* from MNAR to MAR (Figure 1). We cannot prove that *X* is randomly sampled unless we measure some of the unobserved values, but strong correlations, the ability to explain missingness, and domain knowledge may provide evidence that the data are MAR.

Imputation methods assume specific mechanisms of missingness, and assumption violations can lead to bias in the results of downstream analyses that can be difficult to predict [7,8]. Variances of imputed values are often underestimated, causing artificially low *P* values [9]. Additionally, for data MNAR, the observed values have a different distribution from the missing values. To cope with this, a model can be specified to represent the missing data mechanism, but such models can be difficult to evaluate and may have a large impact on results. Great caution should be taken when handling missing data, particularly data that are MNAR. Most imputation methods assume that data are MAR or MCAR, but it is worth reiterating that these are all idealized states, and real data invariably fall somewhere in between (Figure 1).

### Objective

We aimed to provide a framework for characterizing and understanding the types of missing data present in the EHR. We also developed an open source framework that other researchers can follow when dealing with missing data.

## Methods

### Source Code

We provide the source code to reproduce this work in our repository on GitHub (GitHub, Inc) [10] under a permissive open source license. In addition, we used continuous analysis [11] to generate Docker Hub (Docker Inc) images matching the environment of the original analysis and to create intermediate results and logs. These artifacts are freely available [12].

### Electronic Health Record Data Processing

All laboratory assays were mapped to Logical Observation Identifiers Names and Codes (LOINC). We restricted our analysis to outpatient laboratory results to minimize the effects of extreme results from inpatient and emergency department data. We used all laboratory results dated between August 8, 1996 and March 3, 2016, excluding codes for which less than 0.5% of patients had a result. The resulting dataset consisted of 669,212 individuals and 143 laboratory assays.

We removed any laboratory results that were obtained prior to the patient’s 18th birthday or after their 90th. In cases where a date of death was present, we also removed laboratory results that were obtained within 1 year of death, as we found that the frequency of observations often spiked during this period and the values for certain laboratory tests were altered for patients near death. For each patient, a median date of observation was calculated based on their remaining laboratory results. We defined a temporal window of observation by removing any laboratory results recorded more than 5 years from the median date. We then calculated the median result of the remaining laboratory tests for each patient. As each variable had a different scale and many deviated from normality, we applied Box-Cox and Z-transformations to all variables. The final dataset used for all downstream analyses contained 602,366 patients and 146 variables (age, sex, body mass index [BMI], and 143 laboratory measures).

### Variable Selection

We first ranked the laboratory measures by total amount of missingness, lowest to highest. At each rank, we calculated the percentage of complete cases for the set, including all lower-ranked measures. We also built a random forest classifier to predict the presence or absence of each variable. Based on these results and domain knowledge, we selected 28 variables that provided a reasonable trade-off between quantity and completeness and that we deemed to be largely MAR.

### Predicting the Presence of Data

For each clinical laboratory measure, we used the scikit-learn [13] random forest classifier, to predict whether each value would be present. Each laboratory measure was converted to a binary label vector based on whether the measure was recorded. The values of all other laboratory measures, excluding comembers of a panel, were used as the training matrix input to the random forest. This process was repeated for each laboratory test using 10-fold cross-validation. We assessed prediction accuracy by the area under the receiver operating characteristic curve (AUROC) using the trapezoidal rule.

### Sampling of Complete Cases

To generate a set of complete cases that resembled the whole population, we randomly sampled 100,000 patients without replacement. We then matched each of these individuals to the most similar patient who had a value for each of the 28 most common laboratory tests by matching sex and finding the minimal euclidean distance of age and BMI.

### Simulation of Missing Data

Within the sampled complete cases, we selected the data for removal by 4 mechanisms

#### Simulation 1: Missing Completely at Random

We replaced values with NaN (indicator of missing data) at random. We repeated this procedure 10 times each for 10%, 20%, 30%, 40%, and 50% missingness, yielding 50 simulated datasets.

#### Simulation 2: Missing at Random

We selected 2 columns (*A* and *B*) and a quartile. For the values from column *A* within the quartile, we randomly replaced 50% of the values from column *B* with NaN. We repeated the procedure for each quartile and each laboratory test combination, yielding 3024 simulated datasets.

#### Simulation 3: Missing Not at Random

We selected a column and a quartile. When the column’s value was in the quartile, we replaced it with NaN 50% of the time. We repeated this procedure for each of the 4 quartiles of each of the 28 laboratory values, generating a total 112 total simulated datasets.

#### Simulation 4: Missingness Based on Real Data Observations

From our complete-cases dataset, we matched each patient to the nearest neighbor, excluding self-matches, in the entire population based on their sex, age, and BMI. We then replaced any laboratory value in the complete cases with NaN if it was absent in the matched patient.

### Imputation of Missing Data

Using our simulated datasets (simulations 1-4), we compared 18 common imputation methods (12 representative methods are shown in the figures below) from the fancyimpute [14] and the Multivariate Imputation by Chained Equations (MICE v2.30) [15] packages. Multimedia Appendix 1 (table) shows a full list of imputation methods and the parameters used for each.

## Results

**Figure 2:**
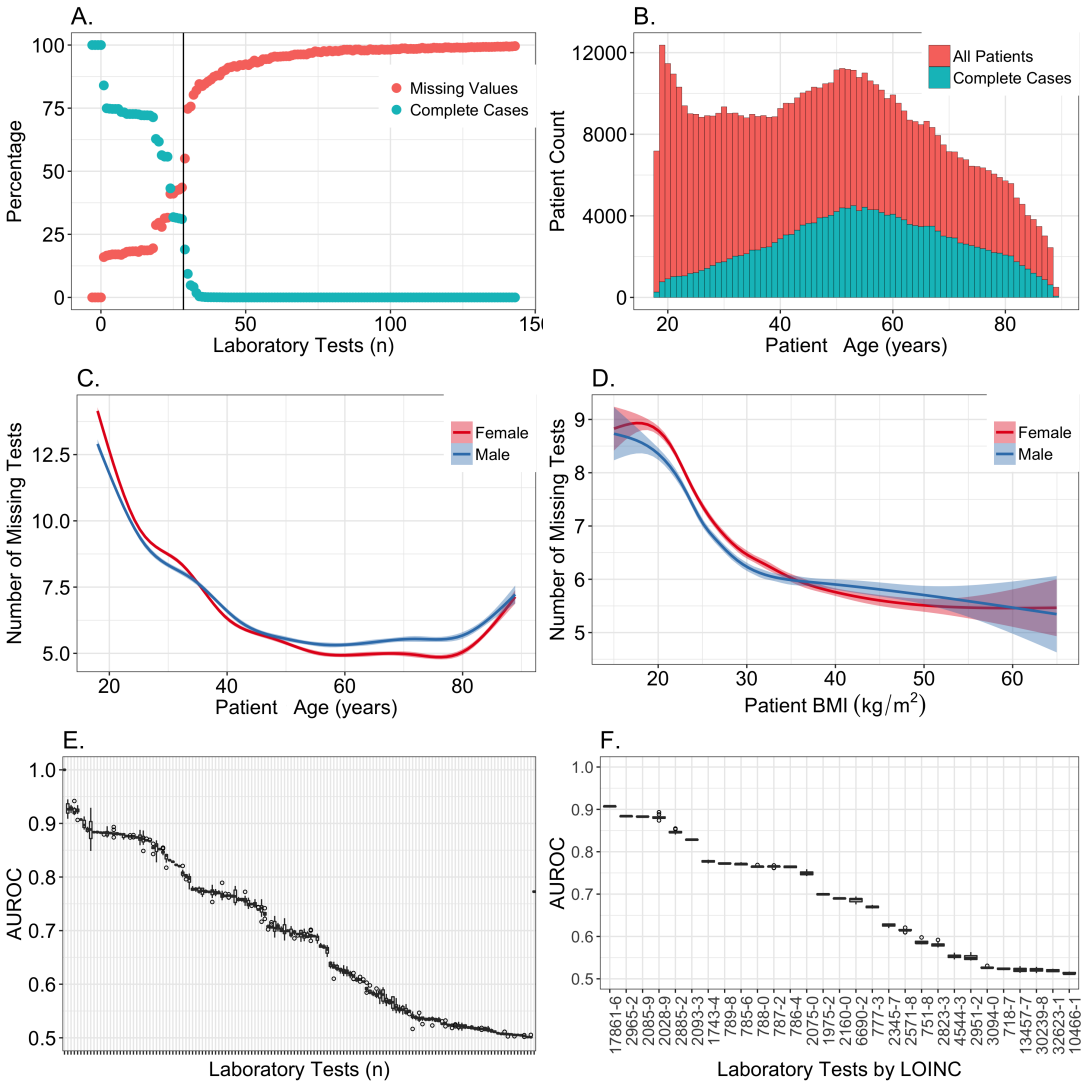
Summary of missing data across 143 clinical laboratory measures. (A) After ranking the clinical laboratory measures by the number of total results, the percentage of patients missing a result for each test was plotted (red points). At each rank, the percentage of complete cases for all tests of equal or lower rank were also plotted (blue points). Only variables with a rank ≤75 are shown. The vertical bar indicates the 28 tests that were selected for further analysis. (B) The full distribution of patient median ages is shown in blue, and the fraction of individuals in each age group that had a complete set of observations for tests 1-28 are shown in red. (C) Within the 28 laboratory tests that were selected for imputation analyses, the mean number of missing tests is depicted as a function of age. (D) Within the 28 laboratory tests that were selected for imputation, the mean number of missing tests is depicted as a function of body mass index (BMI). (E) Accuracy of a random forest predicting the presence or absence of all 143 laboratory tests. AUROC: area under the receiver operating characteristic curve. (F) Accuracy of a random forest predicting the presence or absence of the top 28 laboratory tests, by Logical Observation Identifiers Names and Codes (LOINC).

Our first step was to select a subset of the 143 laboratory measures for which imputation would be a reasonable approach. We began by ranking the clinical laboratory measures in descending order by the number of patients who had an observed value for that test. For each ranked laboratory test, we plotted the percentage of individuals missing a value, as well as the percentage of complete cases when that given test was joined with all the tests with lower ranks (ie, less missingness). These plots showed that the best trade-off between quantity of data and completeness was between 20 and 30 variables (Figure 2, part A). Beyond the 30 most common laboratory tests, the number of complete cases rapidly approached zero.

As age, sex, and BMI have a considerable impact on what clinical laboratory measures are collected, we evaluated the relationship between missingness and these covariates (Figure 2, parts B-D). We also used a random forest approach to predict the presence or absence of each measure based on the values of the other observed measures. MCAR data are not predictable, resulting in AUROCs near 0.5. Only 38 of the 143 laboratory tests had AUROCs less than 0.55 (Figure 2, part E). Very high AUROCs are most consistent with data that are MAR. For the top 30 candidate clinical laboratory measures based on the number of complete cases, the mean AUROC was 0.82. This suggested that the observed data could explain much of the mechanism responsible for the missing data within this set. We ultimately decided not to include the 29th-ranked laboratory test, specific gravity of urine (2965-2), since it had an AUROC of only 0.69 and is typically used for screening only within urology or nephrology departments (RV Levy, MD, personal conversation, June 2017). We included the lipid measures (ranks 25-28), since they had AUROC values near 0.82 and they are recommended for screening of patients depending on age, sex, and BMI [16]. Our data confirmed that age, sex, and BMI all predicted the presence of lipid measures (Multimedia Appendix 1, fig 1A-B).

To assess the accuracy of imputation methods, we required known values to compare with imputed values. Thus, we restricted our analysis to a subset of patients who were complete cases for the 28 selected variables (Table 1) [17]. Since the characteristics of this subset differed from those of the broader population (Figure 2, parts B-D), we used sampling and k-nearest neighbors (KNN) matching to generate a subset of the complete cases that better resembled the overall population. We then simulated missing data within this set by 4 mechanisms: MCAR, MAR, MNAR, and realistic patterns based on the original data.

**Figure 3:**
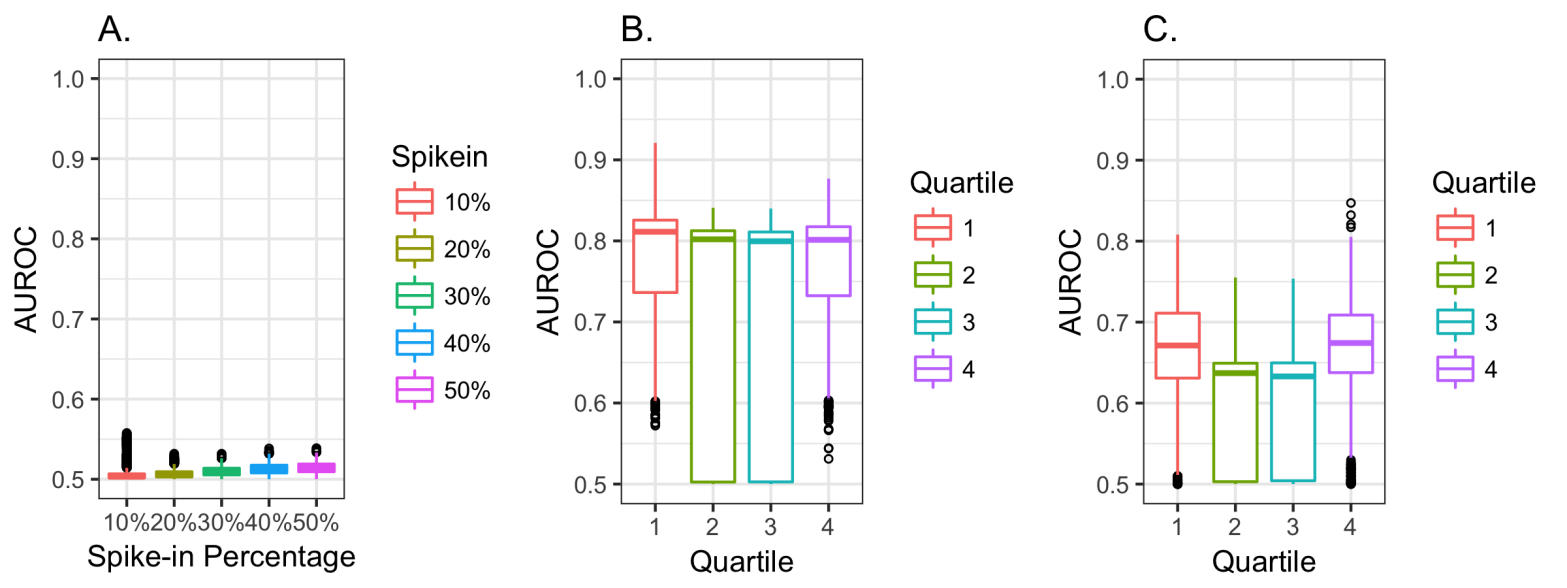
Area under the receiver operating characteristic curve (AUROC) of a random forest predicting whether data will be present or missing. (A) Missing completely at random simulation. (B) Missing at random simulation. (C) Missing not at random simulation.

We next evaluated our ability to predict the presence of each value in the simulated datasets. These simulations confirmed that our MCAR simulation had a low AUROC (Figure 3, part A). The MAR data (Figure 3, part B) and MNAR data (Figure 3, part C) were often well predicted, particularly for the MAR data and when data were missing from the tails of distributions. The AUROCs rarely exceeded 0.75 in the MNAR simulations, while values above 0.75 were typical in the MAR simulations. This provided additional support for our decision to restrict our focus to the top 28 laboratory measures, since they all had AUROCs between 0.9 and 0.75, which was outside the range of MNAR simulations (Figure 2, part F and Figure 3, part C).

We chose to test the accuracy of imputation for several methods from 2 popular and freely available libraries: the MICE package for R and the fancyimpute library for Python. We first applied each of these methods across simulations 1 to 3. For each combination, Figure 4 depicts the overall root mean square errors. Multimedia Appendix 1 (Supplemental Table and Figures 3-21) shows a breakdown of all the methods and parameters.

**Table 1:** Logical Observation Identifiers Names and Codes (LOINC) and descriptions of the most frequently ordered clinical laboratory measurements. The assays are ranked from the most common to the least.

LOINC	Description
718-7	Hemoglobin [Mass/volume] in Blood
4544-3	Hematocrit [Volume Fraction] of Blood by Automated count
787-2	Erythrocyte mean corpuscular volume [Entitic volume] by Automated count
786-4	Erythrocyte mean corpuscular hemoglobin concentration [Mass/volume] by Automated count
785-6	Erythrocyte mean corpuscular hemoglobin [Entitic mass] by Automated count
6690-2	Leukocytes [#/volume] in Blood by Automated count
789-8	Erythrocytes [#/volume] in Blood by Automated count
788-0	Erythrocyte distribution width [Ratio] by Automated count
32623-1	Platelet mean volume [Entitic volume] in Blood by Automated count
777-3	Platelets [#/volume] in Blood by Automated count
2345-7	Glucose [Mass/volume] in Serum or Plasma
2160-0	Creatinine [Mass/volume] in Serum or Plasma
2823-3	Potassium [Moles/volume] in Serum or Plasma
3094-0	Urea nitrogen [Mass/volume] in Serum or Plasma
2951-2	Sodium [Moles/volume] in Serum or Plasma
2075-0	Chloride [Moles/volume] in Serum or Plasma
2028-9	Carbon dioxide, total [Moles/volume] in Serum or Plasma
17861-6	Calcium [Mass/volume] in Serum or Plasma
1743-4	Alanine aminotransferase [Enzymatic activity/volume] in Serum or Plasma by With P-5'-P
30239-8	Aspartate aminotransferase [Enzymatic activity/volume] in Serum or Plasma by With P-5'-P
1975-2	Bilirubin.total [Mass/volume] in Serum or Plasma
2885-2	Protein [Mass/volume] in Serum or Plasma
10466-1	Anion gap 3 in Serum or Plasma
751-8	Neutrophils [#/volume] in Blood by Automated count
2093-3	Cholesterol [Mass/volume] in Serum or Plasma
2571-8	Triglyceride [Mass/volume] in Serum or Plasma
2085-9	Cholesterol in HDL^a^ [Mass/volume] in Serum or Plasma
13457-7	Cholesterol in LDL^b^ [Mass/volume] in Serum or Plasma by calculation

^a^HDL: high-density lipoprotein.

^b^LDL: low-density lipoprotein.

**Figure 4:**
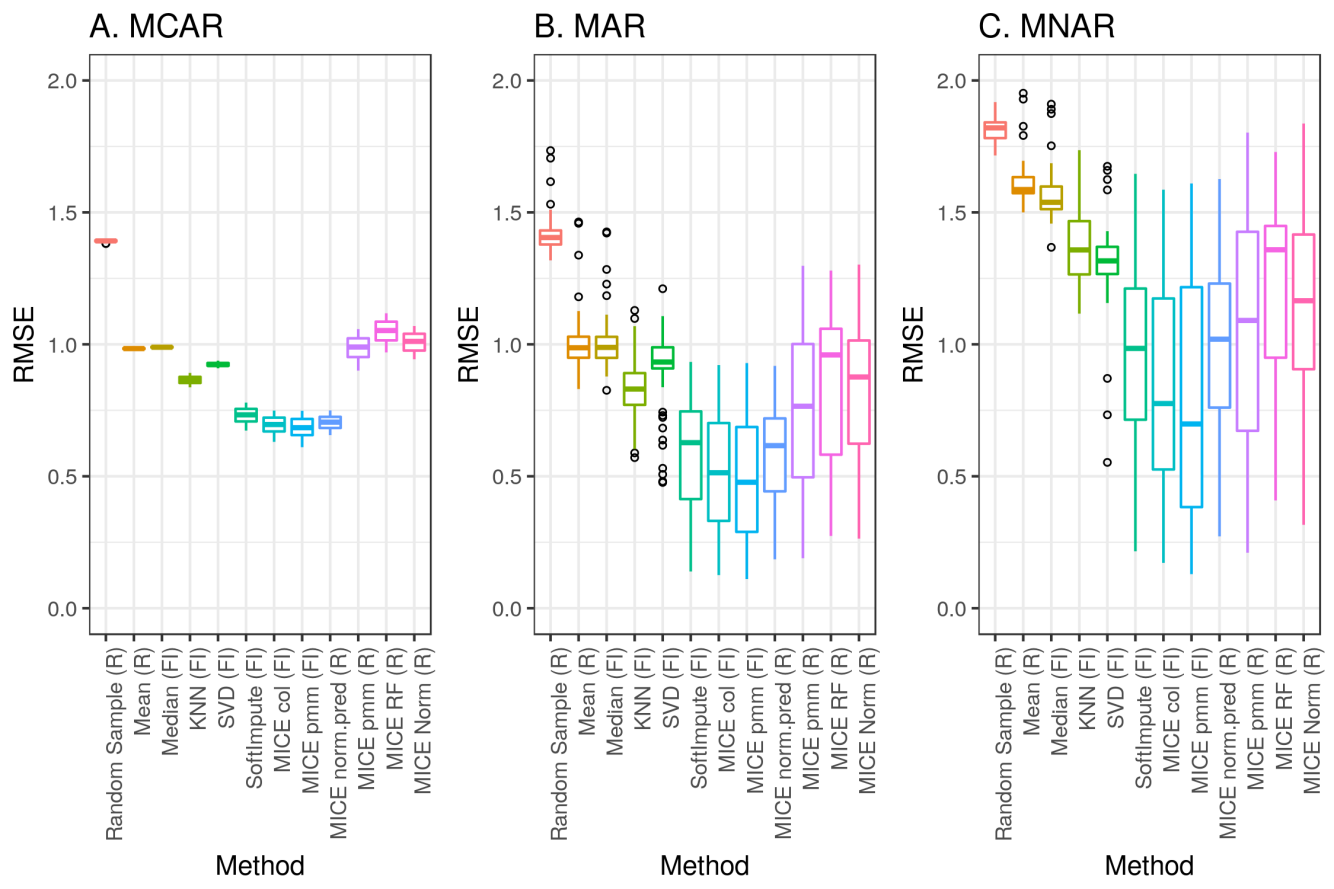
Imputation accuracy measured by root mean square error (RMSE) across simulations 1-3. (A) Missing completely at random (MCAR). (B) Missing at random (MAR). (C) Missing not at random (MNAR). FI: fancyimpute; KNN: k-nearest neighbors; MICE: Multivariate Imputation by Chained Equations; pmm: predictive mean matching; RF: random forest; SVD: singular value decomposition.

**Figure 5:**
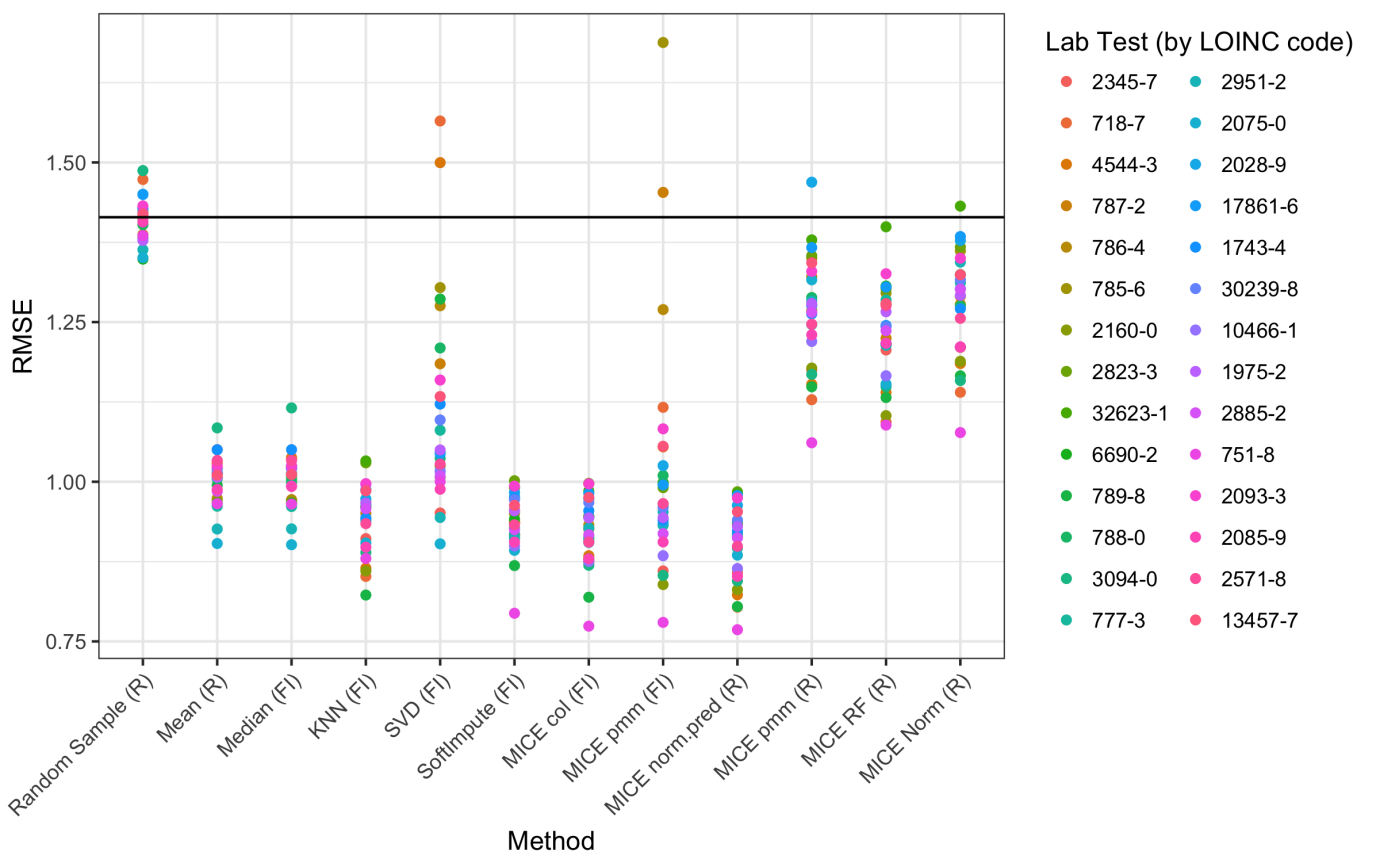
Imputation root mean square error (RMSE) for a subset of 10,000 patients from simulation 4. A total of 12 imputation methods were tested (x-axis), and each color corresponds to a Logical Observation Identifiers Names and Codes (LOINC) code. The black line shows the theoretical error from random sampling. FI: fancyimpute; KNN: k-nearest neighbors; MICE: Multivariate Imputation by Chained Equations; pmm: predictive mean matching; RF: random forest; SVD: singular value decomposition.

We next measured imputation accuracy based on the patterns of missingness that we observed in the real data (Figure 5). The main difference compared with simulations 1 to 3 was lower error for some of the deterministic methods (mean, median, and KNN). It is worth mentioning that the error was highly dependent on the variable that was being imputed. Specifically, for the fancyimpute MICE predictive mean matching (pmm) method, multicollinearity within some of the variables caused convergence failures that led to extremely large errors (Figure 5, method MICE pmm [FI]). These factors were relatively easy to address in the R package MICE pmm method by adjusting the predictor matrix [15].

**Figure 6:**
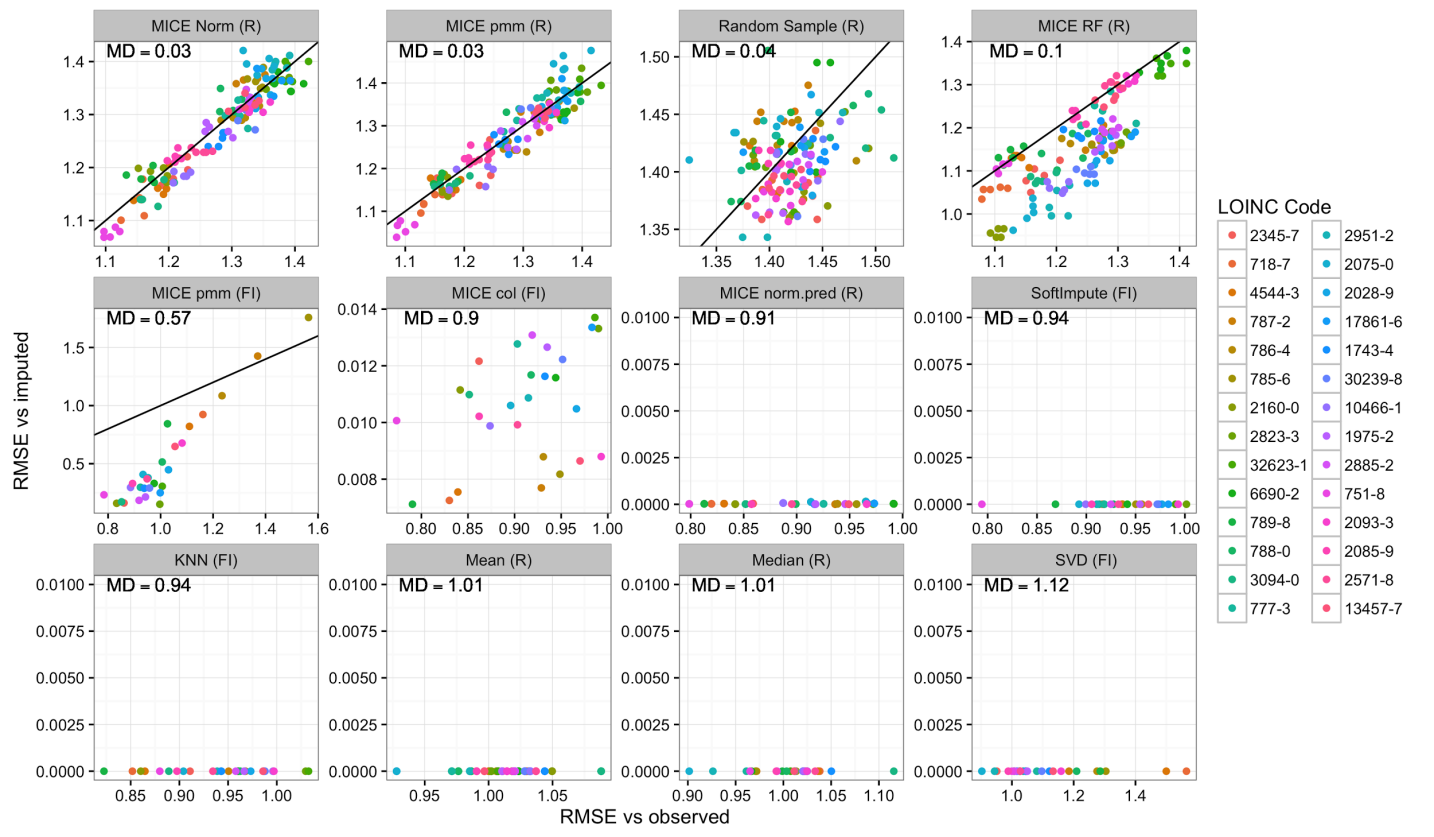
Assessment of multiple imputation for each method. Using simulation 4, missing values were imputed multiple times with each method. The x-axes show the root mean square error (RMSE) between the imputed data and the observed values. The y-axes show the RMSE between multiple imputations of the same data. The axis scales vary between panels to better show the range of variation. The laboratory tests are indicated by the color of the points. The black diagonal line represents unity (y=x). Panels are ordered by each method’s mean deviation (MD) from unity, indicated in the top left corner of each panel. In the last 7 panels, the unity line is not visible because the variation between multiple imputations was close to zero. FI: fancyimpute; KNN: k-nearest neighbors; MICE: Multivariate Imputation by Chained Equations; pmm: predictive mean matching; RF: random forest; SVD: singular value decomposition.

In addition to evaluating the accuracy of imputation, it is also important to estimate the uncertainty associated with imputation. One approach to address this is multiple imputation, where each data point is imputed multiple times using a nondeterministic method. To determine whether each method properly captured the true uncertainty of the data, we compared the error between an imputed dataset and the observed data versus the error between 2 sets of imputed values for each method (Figure 6). If these errors are equal, then multiple imputation is likely producing good estimates of uncertainty. If, however, the error between 2 imputed datasets is less than that between each imputed dataset and the known values, then the imputation method is likely underestimating the variance.

Our results (Figure 6) demonstrate that many of the imputation methods are not suitable for multiple imputation. Of the methods that had the lowest error in the MCAR, MAR, and MNAR simulations we found 3 (softImpute, MICE col (fancyimpute), MICE norm.pred (R)) to have minimal variation between imputations. This was also true of KNN, singular value decomposition (SVD), mean, and median imputation. Only 3 methods (random sampling, MICE norm (R), and MICE pmm (R)) seemed to have similar error between the multiple imputations and the observed data and thus appear to be unbiased. The latter 2 had very similar performance and are the best candidates for multiple imputation. Two methods had intermediate performance. MICE random forest (R) was similar to several other MICE methods in terms of error relative to the observed data, but it produced slightly less variation between each imputed dataset. This seemed to affect some variables more than others but there was no obvious pattern. The MICE pmm (fancyimpute) was not deterministic but it did seem to achieve low error at the expense of increased bias. In this case, the variables that could be imputed with the lowest error also seemed to have the most bias. Since this method claims to be a reimplementation of the MICE pmm (R) method, this may be due to multicollinearity among the variables that could not easily be accounted for, as there was no simple way to alter the predictor matrix.

## Discussion

### Principal Results

It is not possible, or even desirable, to choose “the best” imputation method. There are many considerations that may not be generalizable between different sets of data; however, we can draw some general conclusions about how different methods compare in terms of error, bias, complexity, and difficulty of implementation. Based on our results, there seem to be 3 broad categories of methods.

The first category is the simple deterministic methods. These include mean or median imputation and KNN. While easy to implement, mean or median imputation may lead to severe bias and large errors if the unobserved data are more likely to come from the tails of the observed distribution (Figure 4, parts A-C, methods mean, median, and KNN). This will also cause the variance of the distribution to be underestimated if more than a small fraction of the data is missing. Since these methods are deterministic, they are also not suitable for multiple imputation (Figure 6, bottom row).

KNN is a popular choice for imputation that has been shown to perform very well for some types of data [18,19], but it was not particularly well suited for our data, regardless of the choice of k. This may be due to issues of data dimensionality [20] or to individuals not falling into well-separated groups based on their clinical laboratory results. This method is also not suitable for large datasets, since a distance matrix for all pairs of individuals is stored in memory during computation, and the size of the distance matrix scales with n^2^.

The second category of algorithms could be called the sophisticated deterministic methods. These include SVD, softImpute, MICE col, and MICE norm.pred. SVD performed poorly compared with its counterparts and sometimes produced errors greater than simple random sampling (Figure 5, method SVD). The reasons for this are not clear, but we cannot recommend this method. SoftImpute, MICE col, and MICE norm.pred were among the lowest-error methods in all of our simulations (Figure 5, methods MICE col and norm.pred). The main limitation of these methods is that they cannot be used for multiple imputation (Figure 6, middle row).

The third broad category of algorithms comprises the stochastic methods, which included random sampling and most of the remaining methods in the MICE library. Random sampling almost always produced the highest error (Figure 4 and Figure 5, method random sample), but it has the advantage of being easy to implement and it requires no parameter selection. The MICE methods based on pmm, random forests, and Bayesian linear regression tended to perform similarly in terms of error in most of our simulations (Figure 4 and Figure 5, methods MICE pmm, RF, and norm).

Imputation methods that involve stochasticity allow for a fundamentally different type of analysis called multiple imputation. In this paradigm, multiple imputed datasets (a minimum of 3 and often 10-20 depending on the percentage of missing data) [21-23] are generated, and each is analyzed in the same way. At the end of all downstream analyses, the results are then compared. Typically, the ultimate result of interest is supported by a *P* value, a regression coefficient, an odds ratio, etc. In the case of a multiply imputed dataset, the researcher will have several output statistics that can be used to estimate a confidence interval for the result.

Multiple imputation has been gaining traction recently, and the MICE package has become one of the most popular choices for implementing this procedure. This package is powerful and very well documented [15] but, like all methods for imputation, caution must be exercised. In MICE, each variable is imputed one by one. This entire process is then repeated for a number of iterations such that the values imputed in 1 iteration can update the estimates for the next iteration. The result is a chain of imputed datasets, and this entire process is typically performed in parallel so that multiple chains are generated.

In MICE, several choices must be made. The first obvious choice is the imputation method (ie, equation). Many methods are available in the base package, additional methods can be added from other packages [24], and users can even define their own. We thoroughly evaluated 3 methods in the context of our dataset: pmm, Bayesian linear regression (norm), and random forest.

The pmm is the default choice, and it can be used on a mixture of numeric and categorical variables. We found pmm to have a good trade-off between error and bias, but for our dataset it was critical to remove several variables from the predictor matrix due to strong correlations (*R*>.85) and multicollinearity. Bayesian regression performed similarly but was less sensitive to these issues. If a dataset contains only numeric values, Bayesian regression may be a safer option. Random forest tended to produce results that were slightly biased for a subset of the variables without an appreciable reduction in error. Aside from random sampling, none of the other methods we evaluated were suitable for multiple imputation (Figure 6).

### Conclusions

Many factors must be considered when analyzing a dataset with missing values. This starts by determining whether each variable should be considered at all. Two good reasons to reject a variable are if it has too many missing values or if it is likely to be MNAR. If a variable is deemed to be MNAR, it may still be possible to impute, but the mechanism of missingness should be explicitly modeled, and a sensitivity analysis is recommended to assess how much impact this could have on the final results [25,26]. While a statistical model of the mechanism of missingness is useful, there is no substitute for a deep familiarity with the data at hand and how they were generated.

Having selected the data, one must select an imputation method. Ideally, several methods should be tested in a realistic setting. Great care should be taken to construct a set of complete data that closely resemble all of the relevant characteristics of the data that one wishes to impute. Similar care should then be taken to remove some of these data in ways that closely resemble the observed patterns of missingness. If this is not feasible, one may also simulate a variety of datasets representing a range of possible data structures and missingness mechanisms. Any available imputation methods can then be applied to the simulated data, and error between the imputed data and their known values provide a metric of performance.

While the minimization of error is an important goal, a singular focus on this objective is likely to lead to bias. For each missing value, it is also important to estimate the uncertainty associated with it. This can be achieved by multiple imputation using an algorithm that incorporates stochastic processes. Multiple imputation has become the field standard because it provides confidence intervals for the results of downstream analyses. One should not naively assume that any stochastic process is free of bias. It is important to check that multiple imputation is providing variability that corresponds to the actual uncertainty of the imputed values using a set of simulated data.

## Appendix

Multimedia Appendix 1 Supplemental table and figures.

## Abbreviations

AUROC: area under the receiver operating characteristic curve
BMI: body mass index
EHR: electronic health record
KNN: k-nearest neighbors
LOINC: Logical Observation Identifiers Names and Codes
MAR: missing at random
MCAR: missing completely at random
MICE: Multivariate Imputation by Chained Equations
MNAR: missing not at random
pmm: predictive mean matching
SVD: singular value decomposition
