# Supplementary material for: Characterizing and Managing Missing Structured Data in Electronic Health Records: Data Analysis
Source: JMIR Med Inform. 2018 Feb 23;6(1):e11. doi: 10.2196/medinform.8960 (PMC5845101; doi:10.2196/medinform.8960)
Supplement: Multimedia Appendix 1 [file medinform_v6i1e11_app1.pdf]

Supplementary Information

Method	Source	Parameters
Mean	fancyimpute	
Median	fancyimpute	
Mean	fancyimpute/R MICE	
Random Sampling	R MICE	
K-Nearest Neighbors	fancyimpute	1, 3, 9, 15, 30, 81, 243, 751, 2000, and 6000 neighbors
Singular Value Decomposition	fancyimpute	All ranks from 1 to 24
SoftImpute	fancyimpute	Ranks 0.5, 1, 2, 4, 8, 16, 32, 64 and 128
MICE		
	R MICE	
		pmm - predictive mean matching
		norm - bayesian linear regression analysis
		norm.nob - non-bayesian linear regression
		norm.boot - linear regression with bootstrapping
		norm.predict - predicted value from a linear regression
		mean - mean imputation, control to fancyimpute mean
		rf - random forest
		ri- random indicator
		sample - random sample from observed data, control
	fancyimpute	
		col (posterior predictive distribution), lambda values of 0.0001, 0.001, 0.01, 0.1, 0.25
		pmm (predictive mean matching), 5 nearest neighbors, lambda values of 0.0001, 0.001, 0.01, 0.1, 0.25

Supplementary Table 1. Imputation methods, package sources and parameters used.

Supplemental Figure 1. Additional Summary of missing data across clinical lab measures.

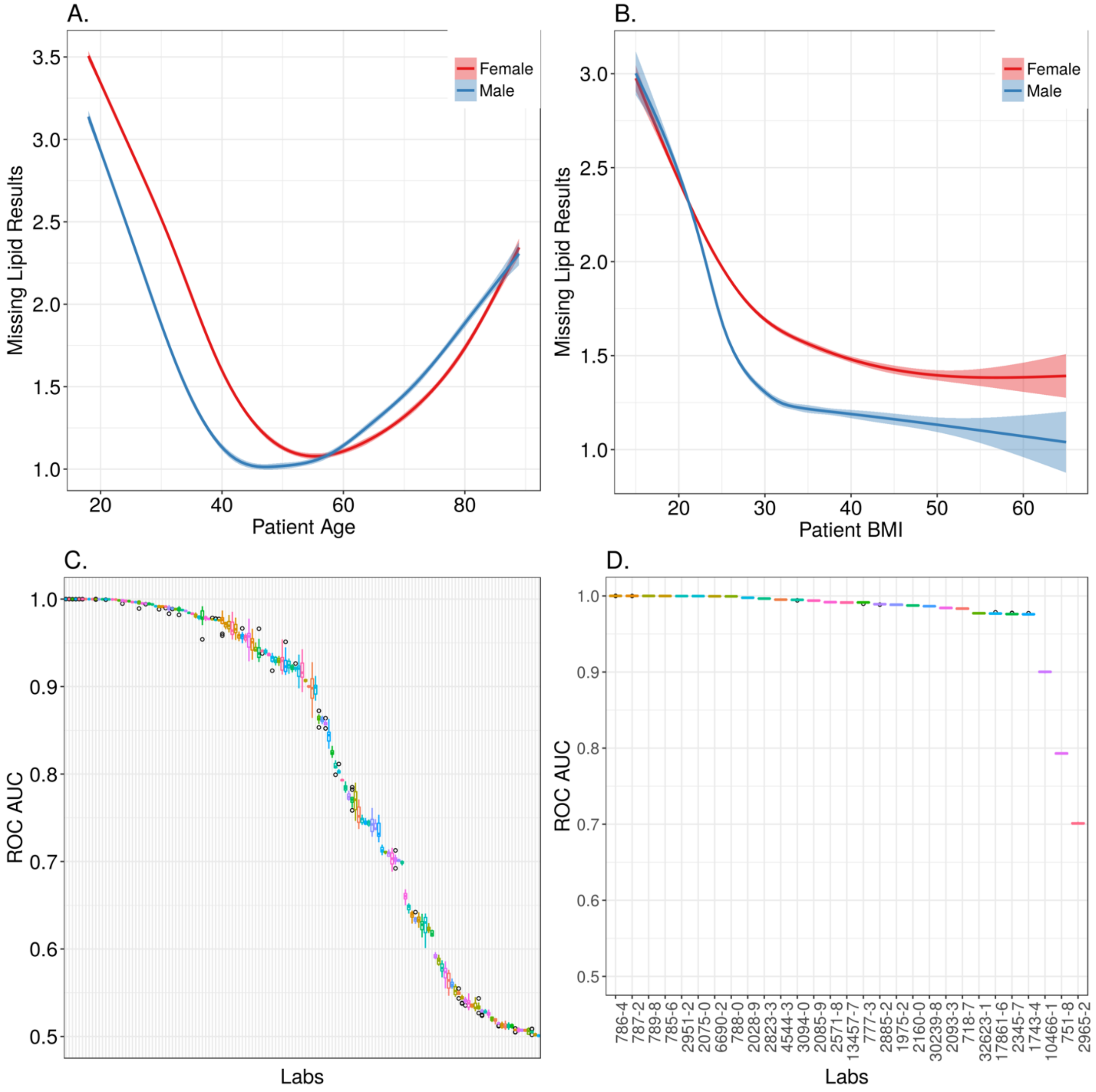

A.) B.) C.) Accuracy of a random forest predicting presence or absence of all 143 laboratory tests. **D.)** Accuracy of a random forest predicting presence or absence of top 28 laboratory tests.

Supplemental Figure 2. Correlation Structure of Variables.

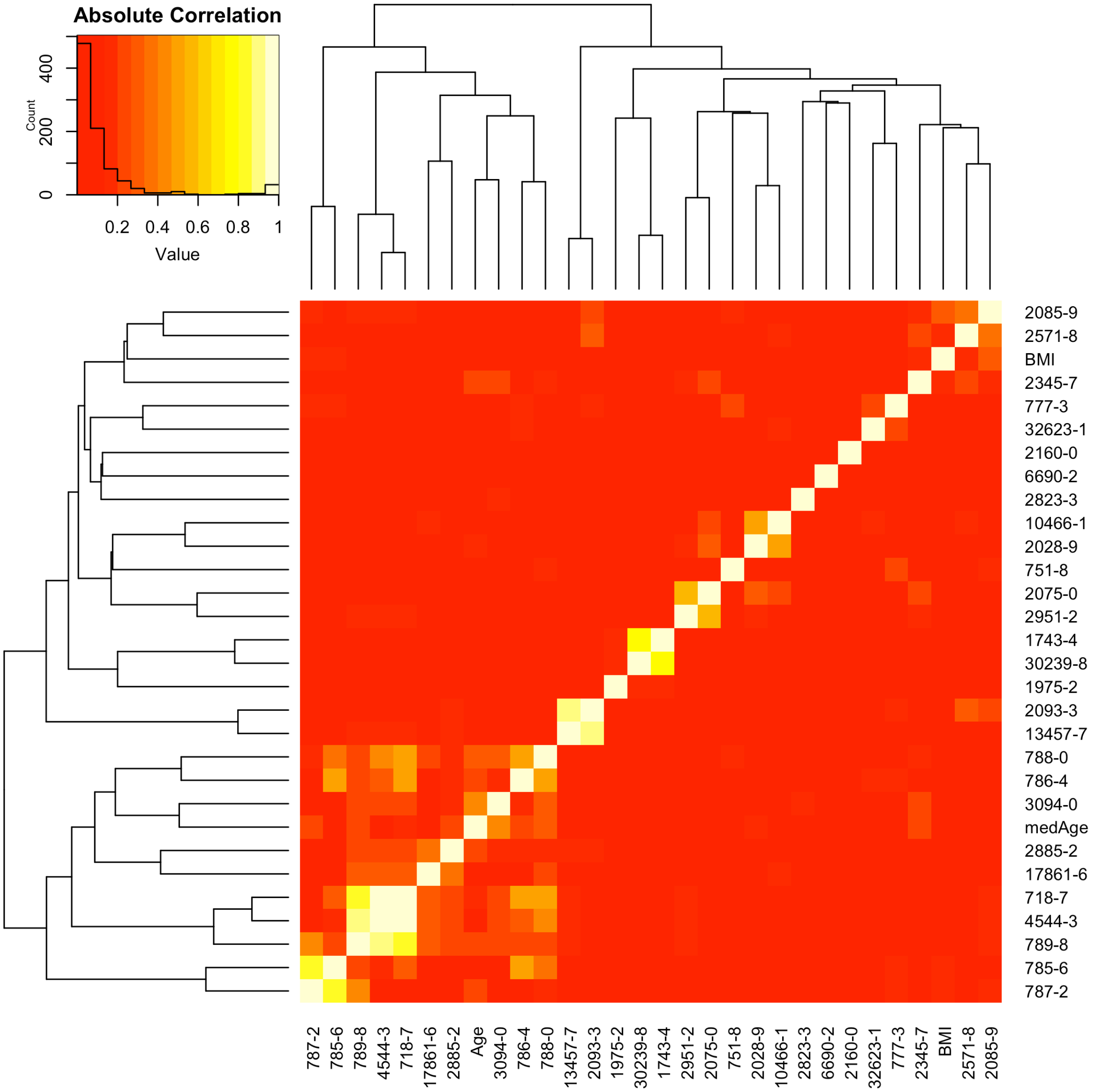

Heatmap of the absolute values of the coefficients of correlation for each pair of variables used in for imputation. Some of the imputation methods were sensitive to multicolinearity and we found that an absolute correlation greater than 0.8 was a sufficient proxy of multicolinearity. There were 4 groups of variable with correlations in this range ({787-2, 785-6}; {789-8, 4544-3, 718-7}; {2093-3, 1975-2}; {1743-4, 30239-8}). In each of these cases, imputation required that only one variable from each of these groups be used to impute other values.

Supplemental Figure 3. KNN MCAR parameter sweep (K values shown).

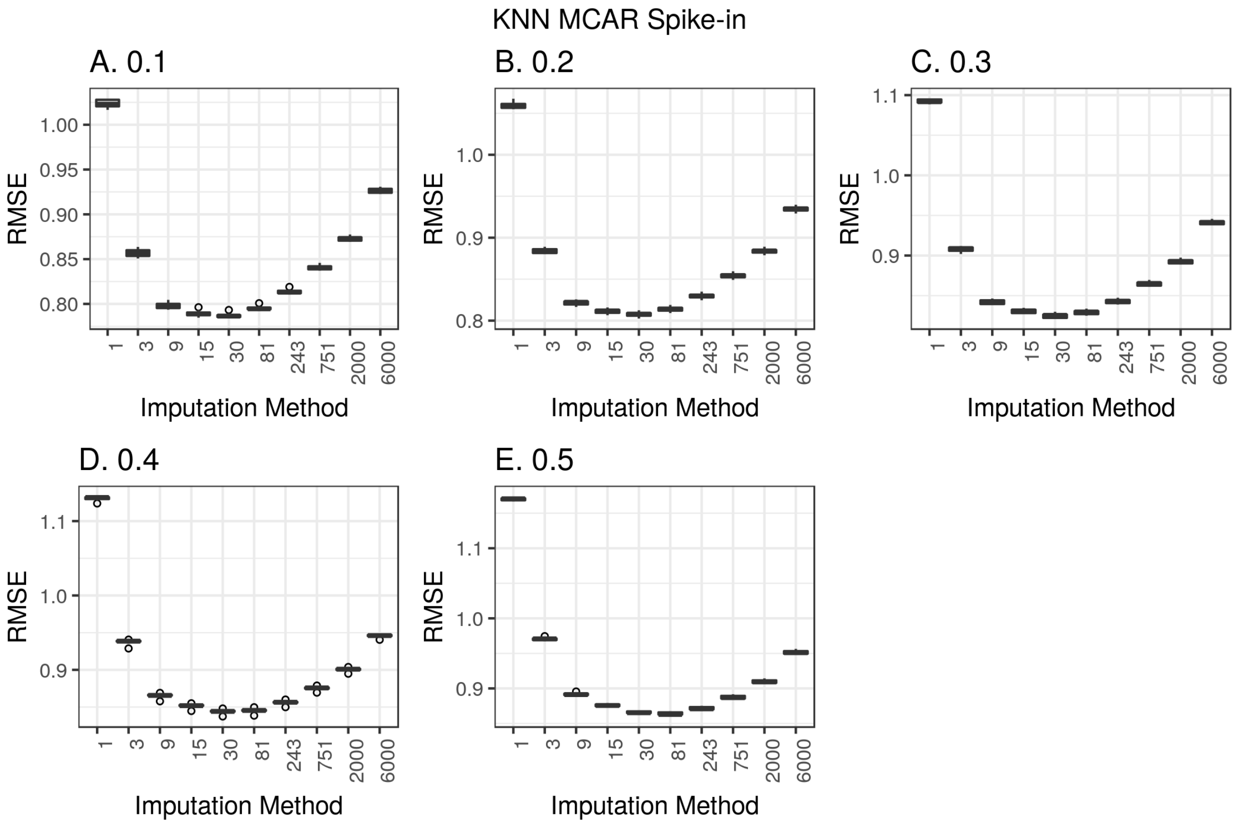

Supplemental Figure 4. Singular Value Decomposition MCAR parameter sweep (Rank values shown).

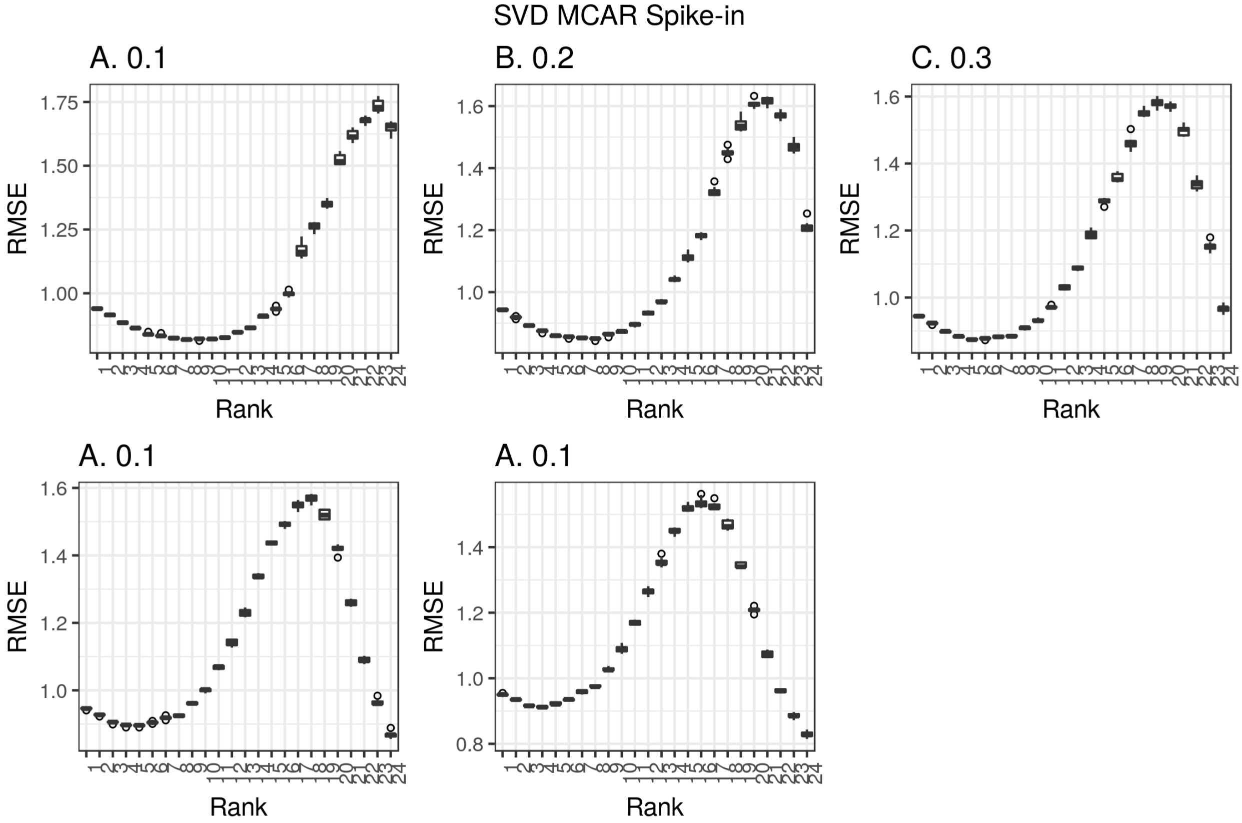

Supplemental Figure 5. SoftImpute MCAR parameter sweep (Shrinkage values shown).

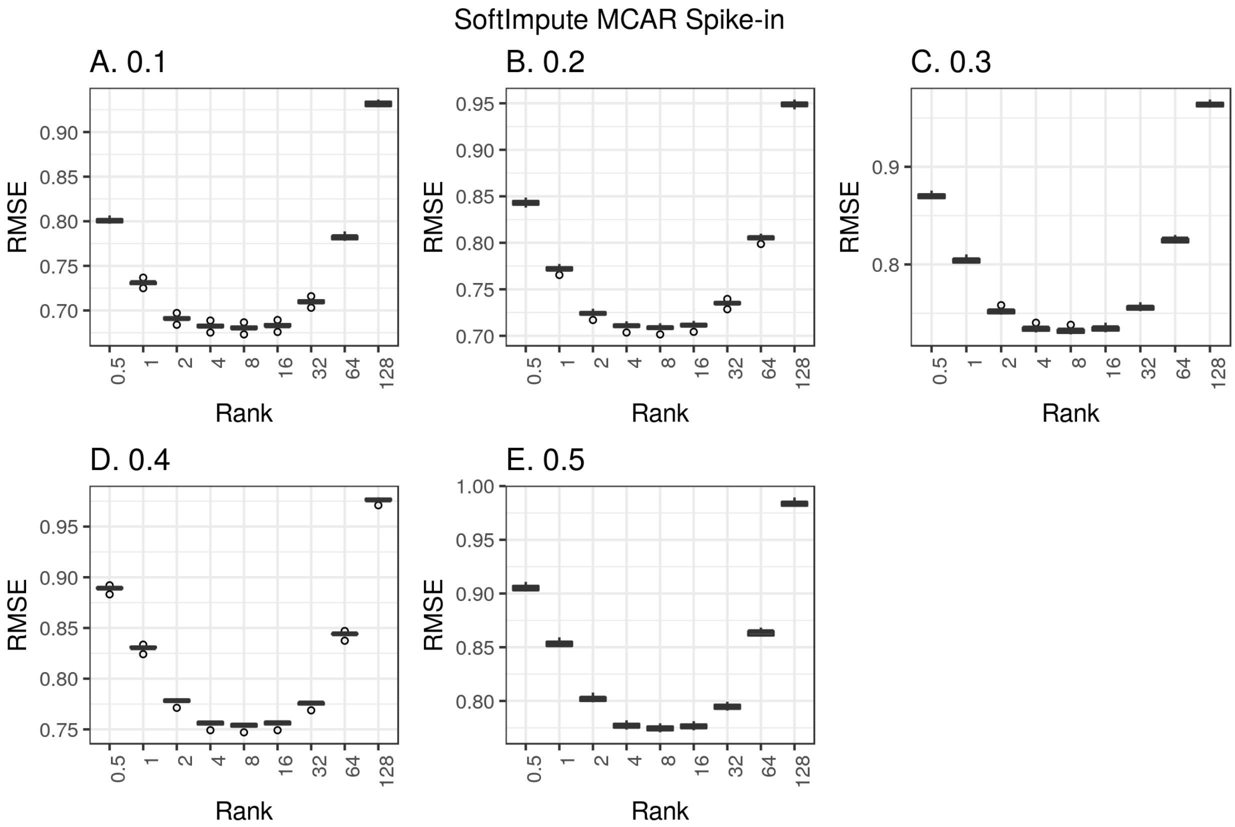

Supplemental Figure 6. MICE Posterior Moment Matching MCAR Spike-in.

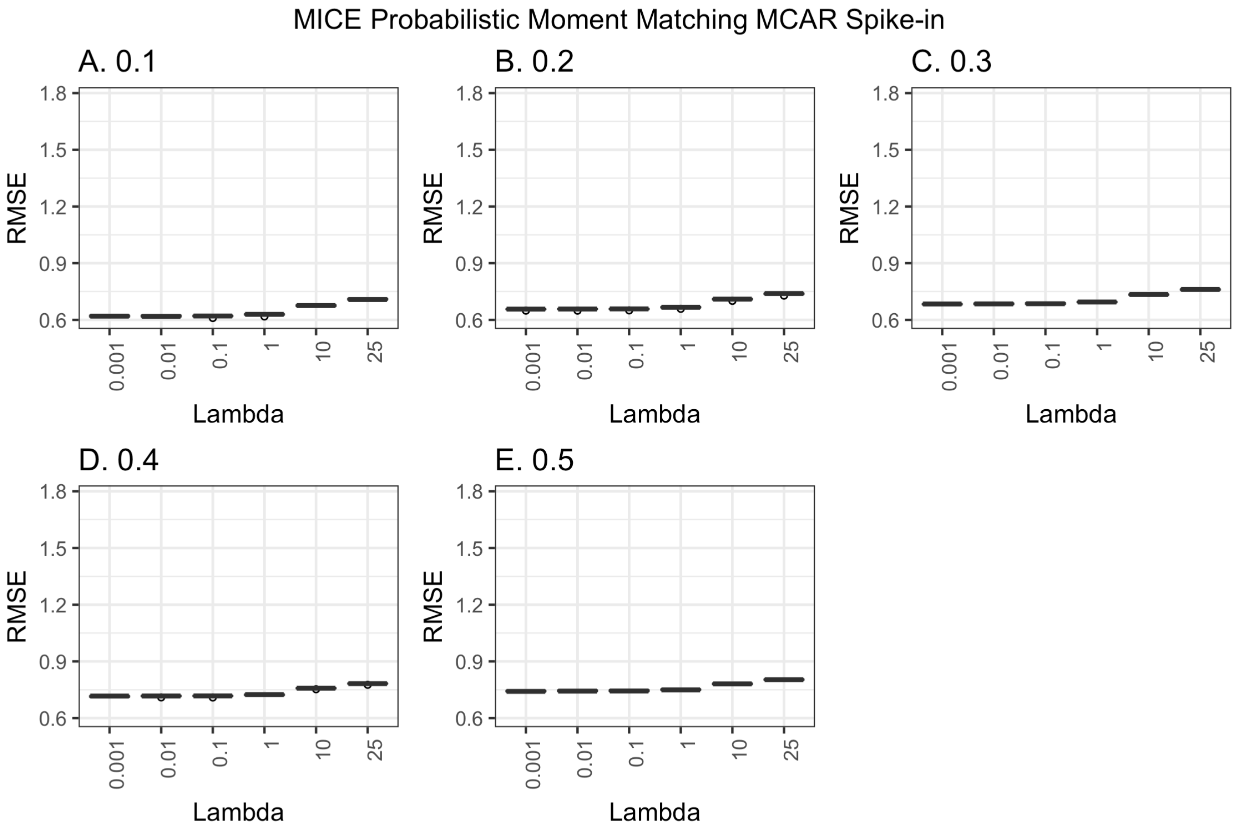

Supplemental Figure 7. MICE Probabilistic Moment Matching MCAR Spike-in.

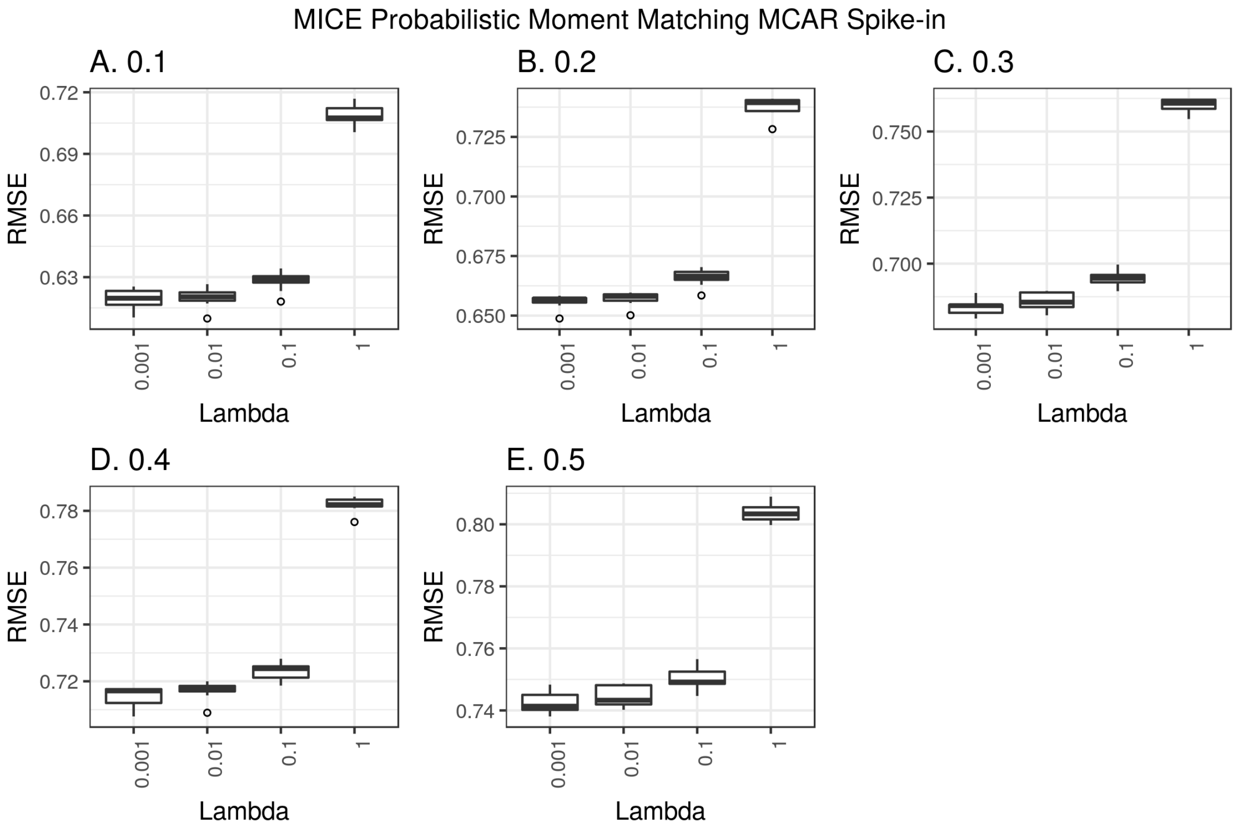

Supplemental Figure 8. MICE R package Spike-in.

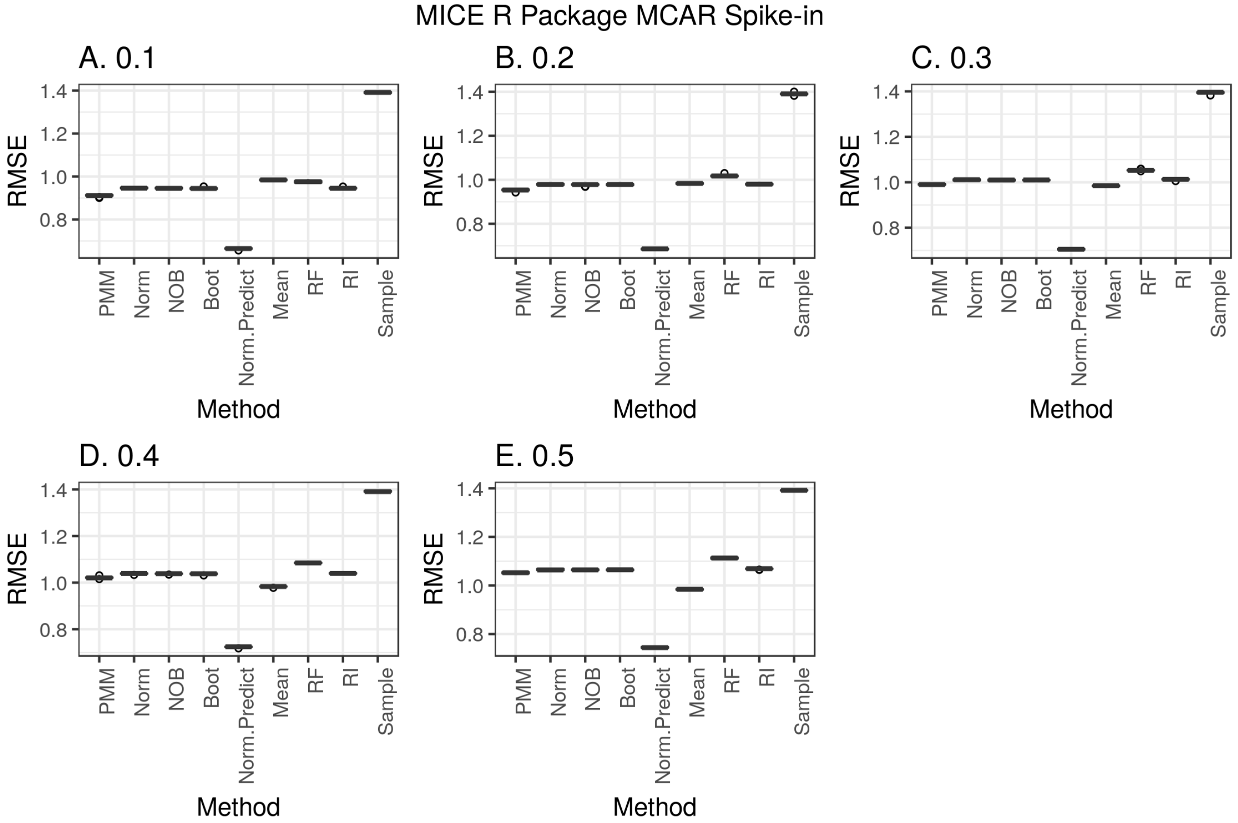

Supplemental Figure 9. KNN MNAR parameter sweep (K values shown).

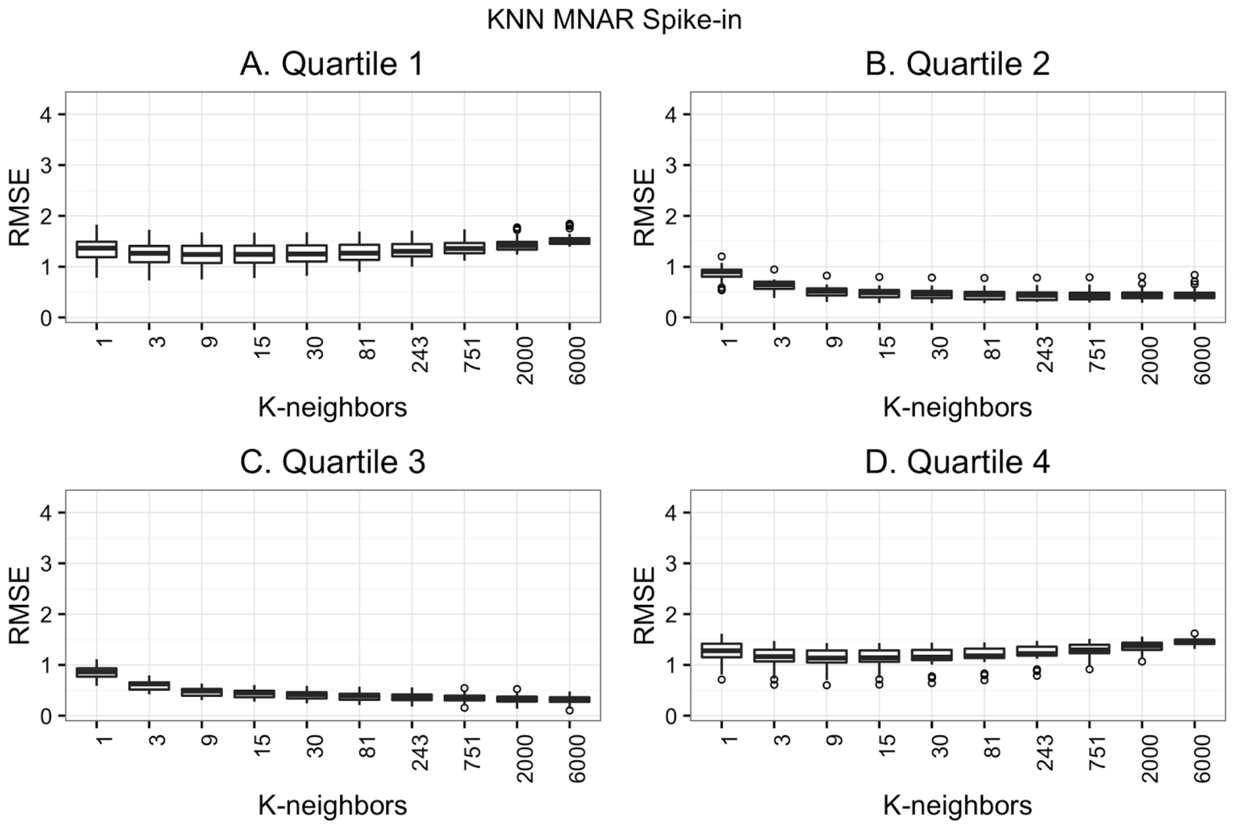

Supplemental Figure 10. Singular Value Decomposition MNAR parameter sweep (Rank values shown).

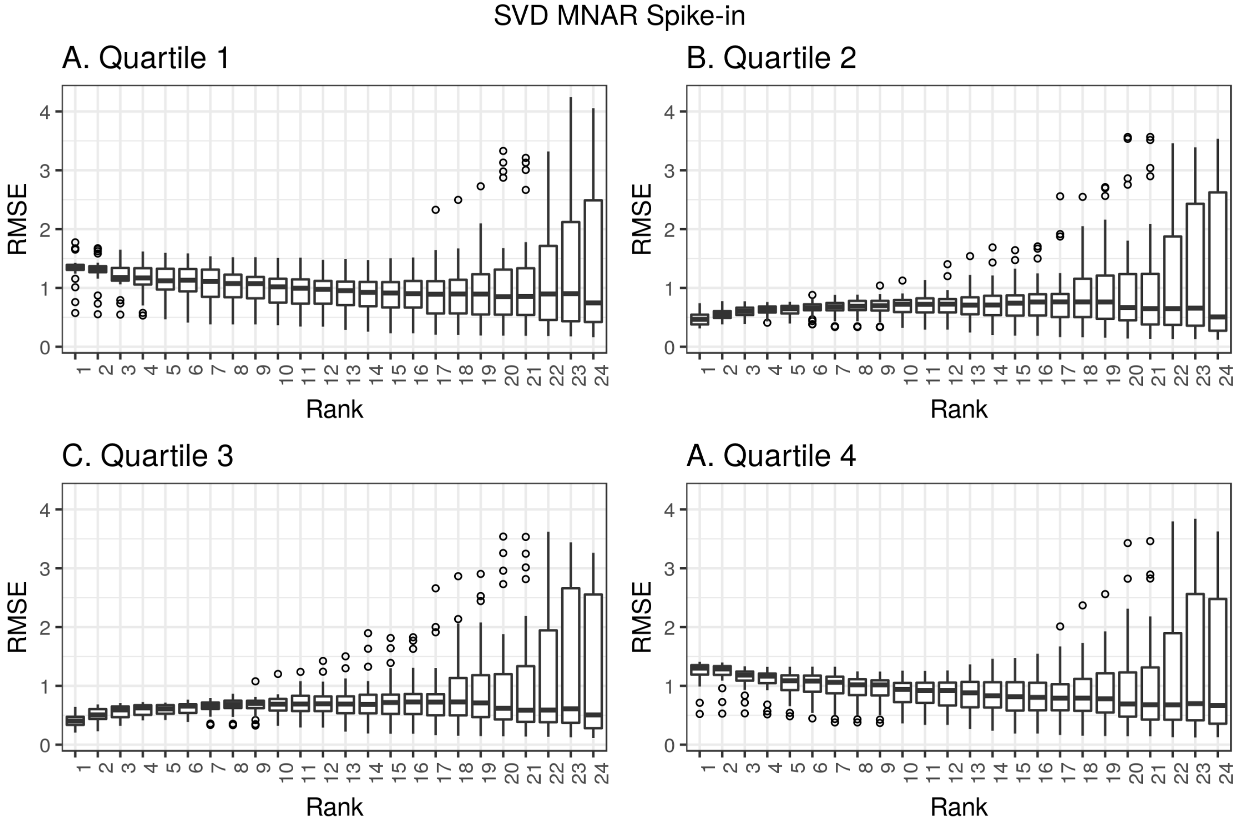

Supplemental Figure 11. SoftImpute MNAR parameter sweep (Shrinkage values shown).

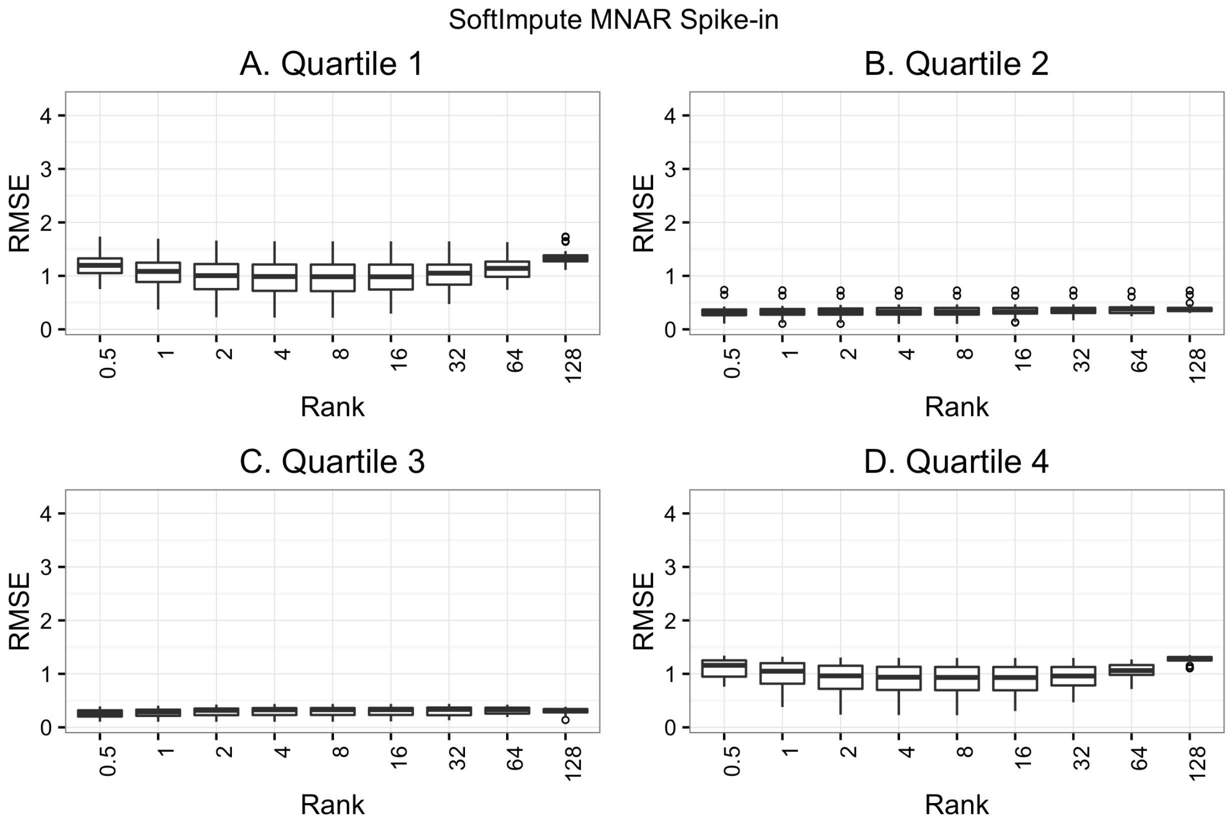

Supplemental Figure 12. MICE Posterior Prediction MNAR Spike-in.

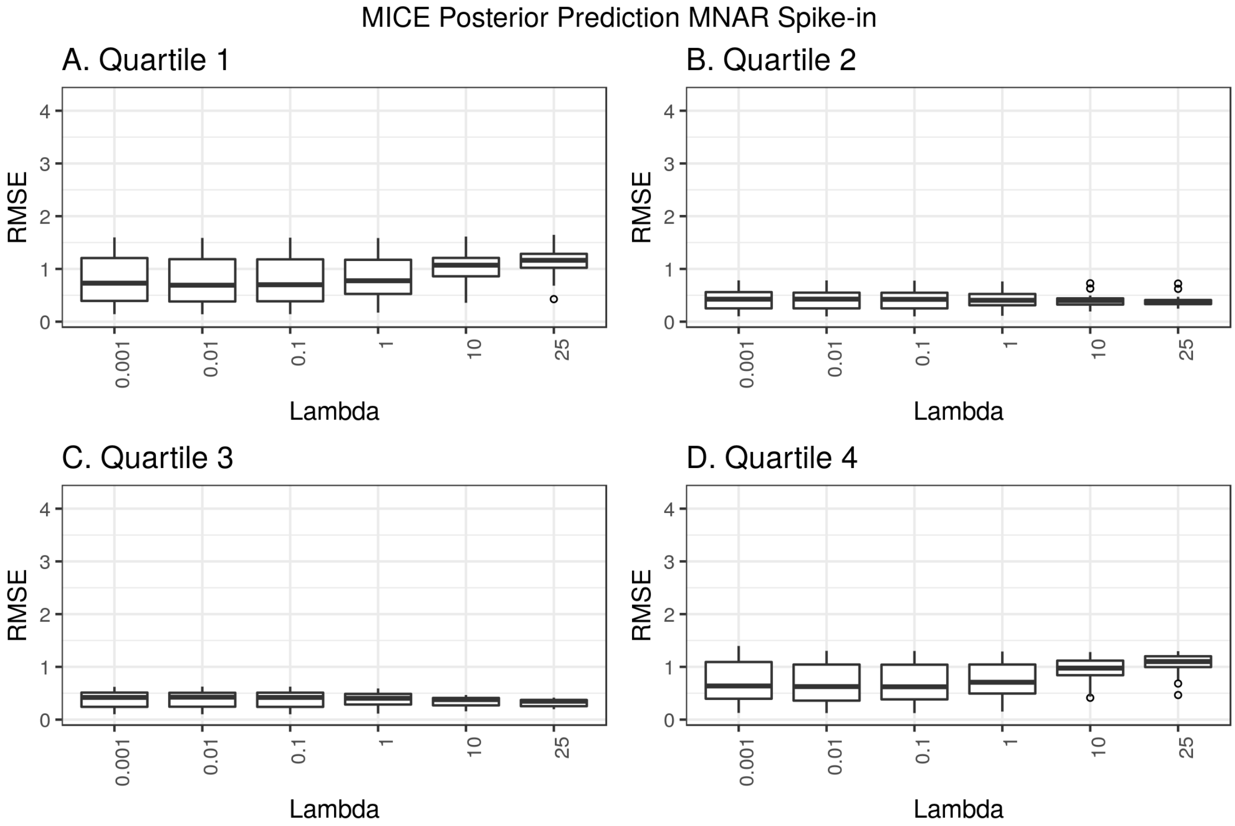

Supplemental Figure 13. MICE Probabilistic Moment Matching MNAR Spike-in.

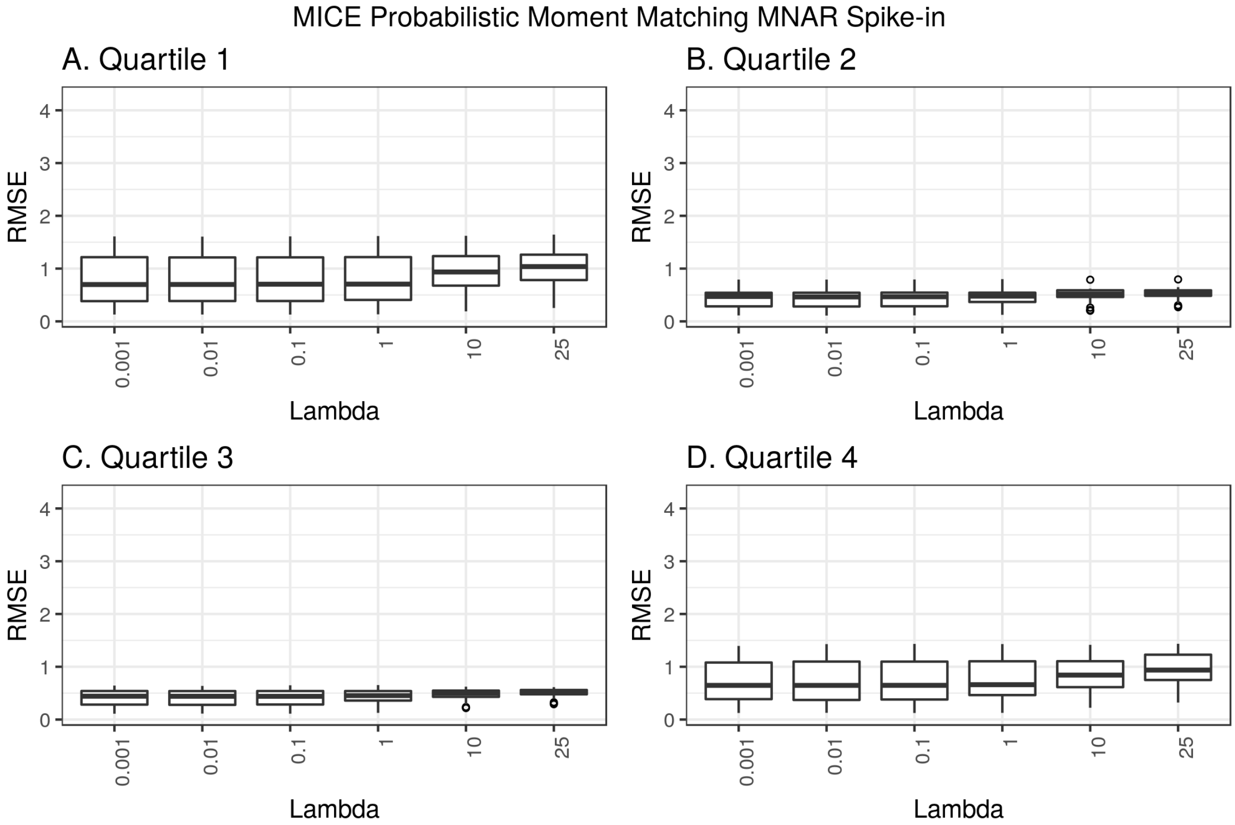

Supplemental Figure 14. MICE R package MNAR Spike-in.

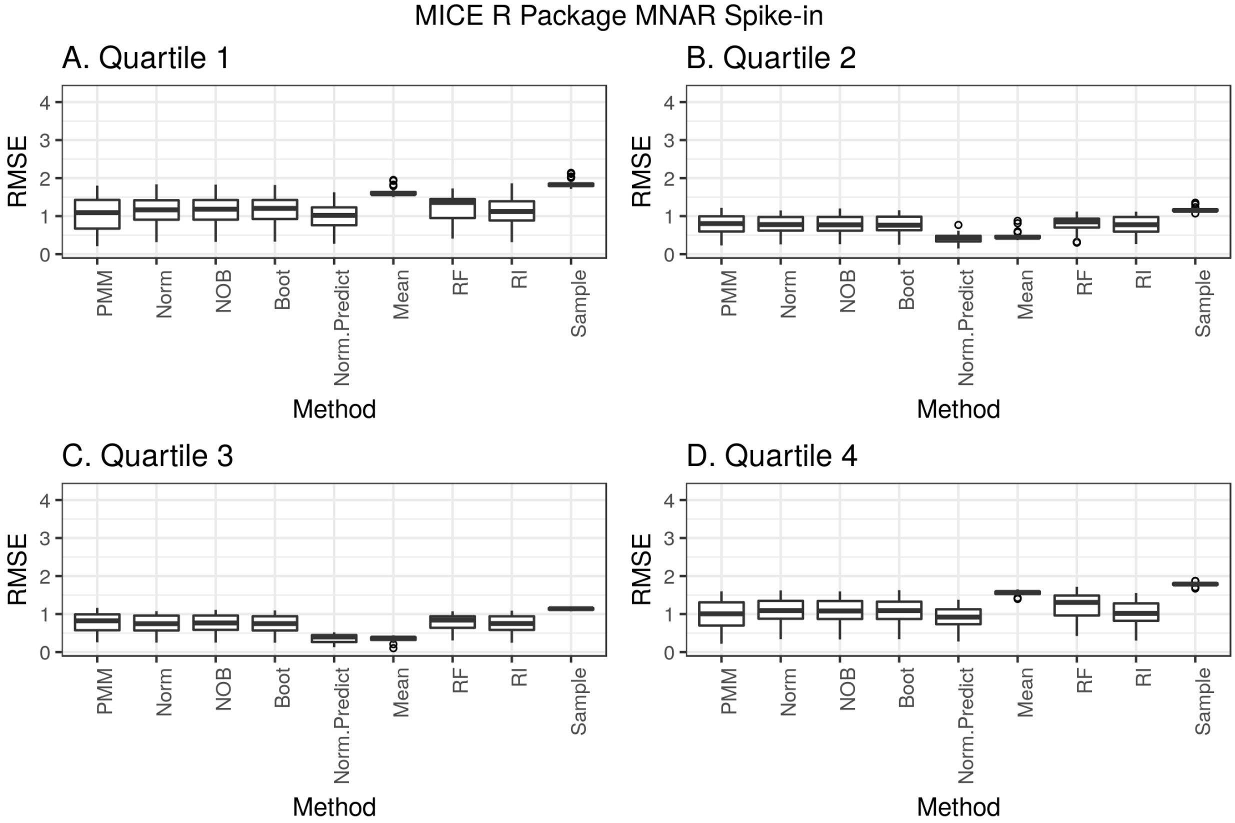

Supplemental Figure 15. KNN MAR parameter sweep (K values shown).

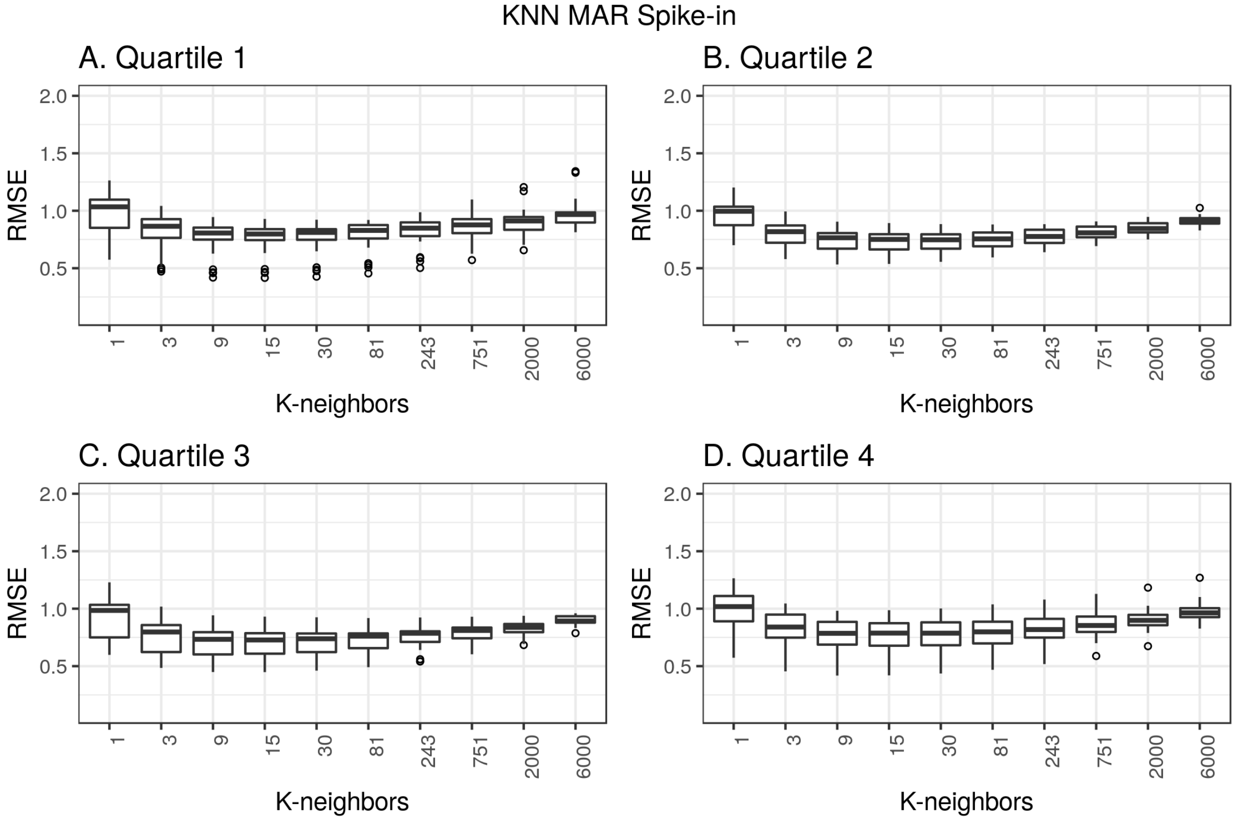

Supplemental Figure 16. Singular Value Decomposition MNAR parameter sweep (Rank values shown).

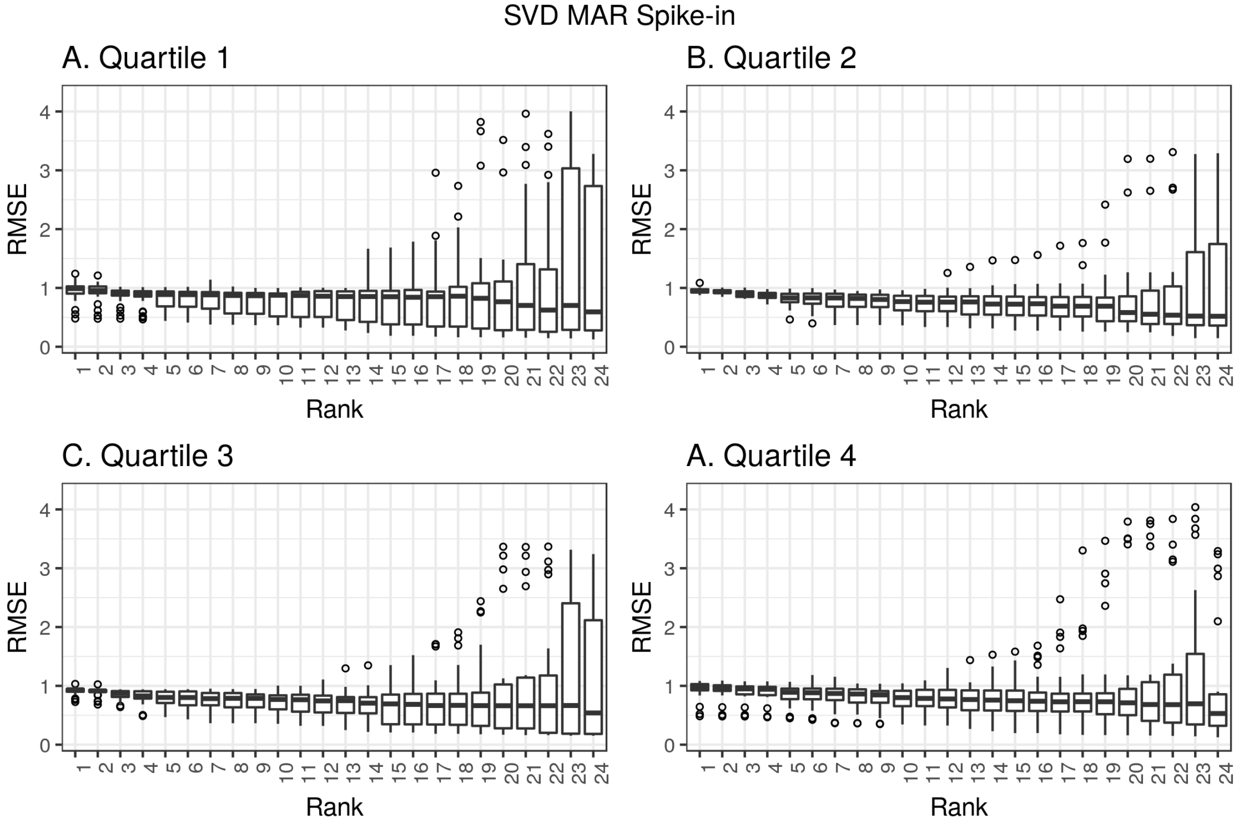

Supplemental Figure 17. SoftImpute MAR parameter sweep (Shrinkage values shown).

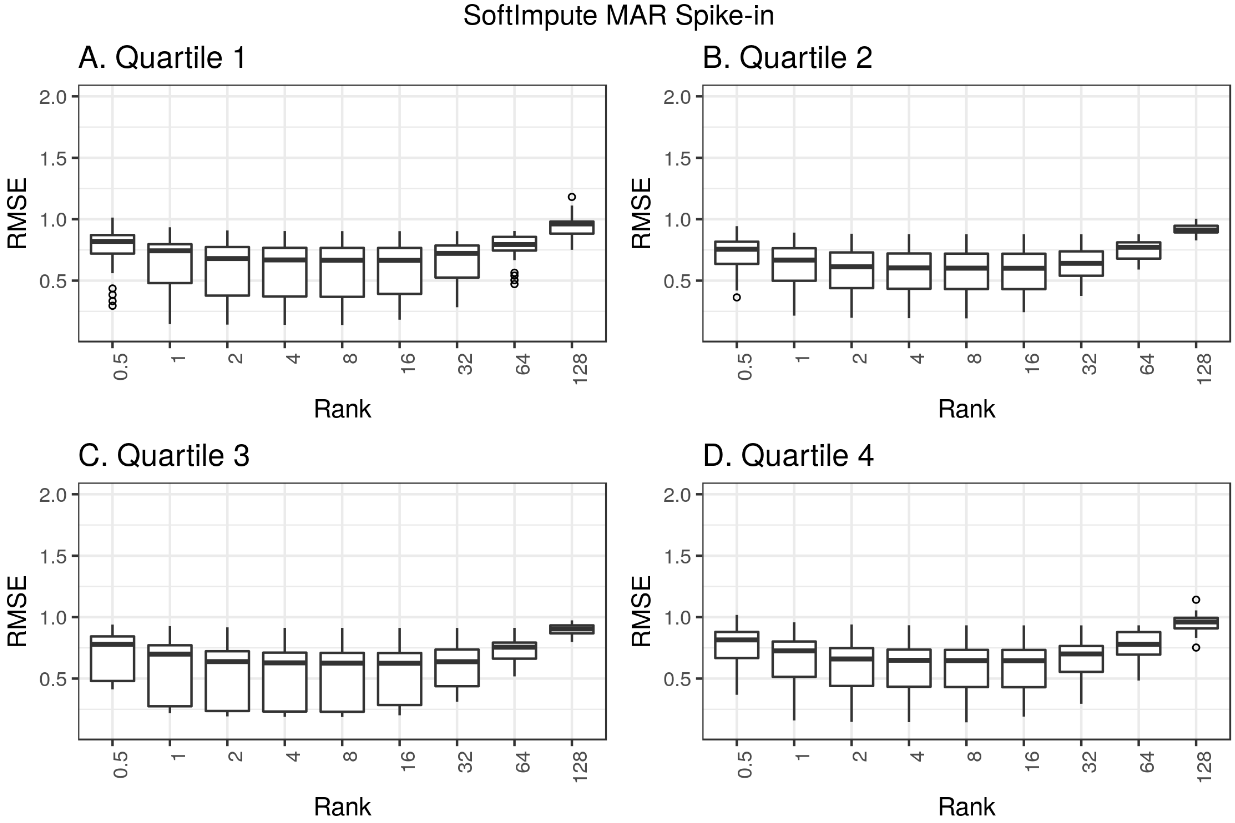

Supplemental Figure 18. MICE Posterior Prediction MAR Spike-in.

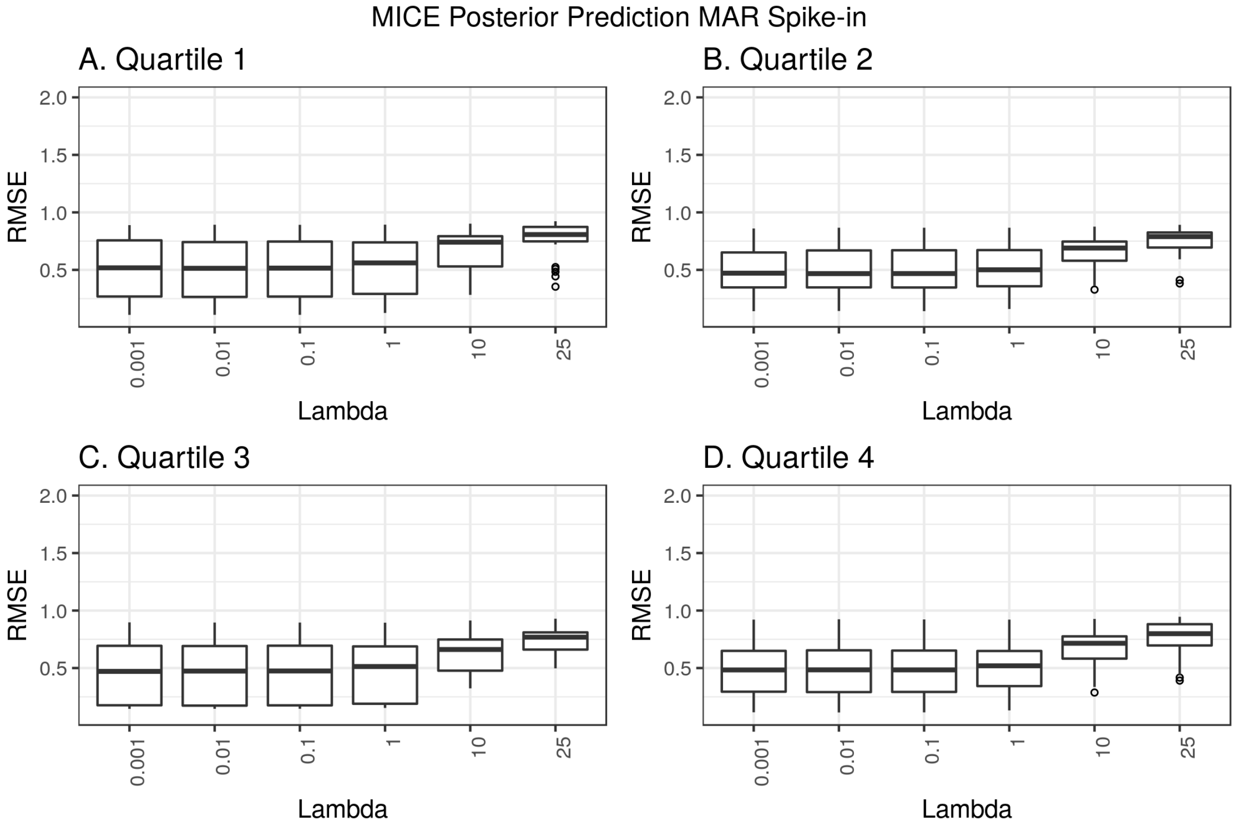

Supplemental Figure 19. MICE Probabilistic Moment Matching MAR Spike-in.

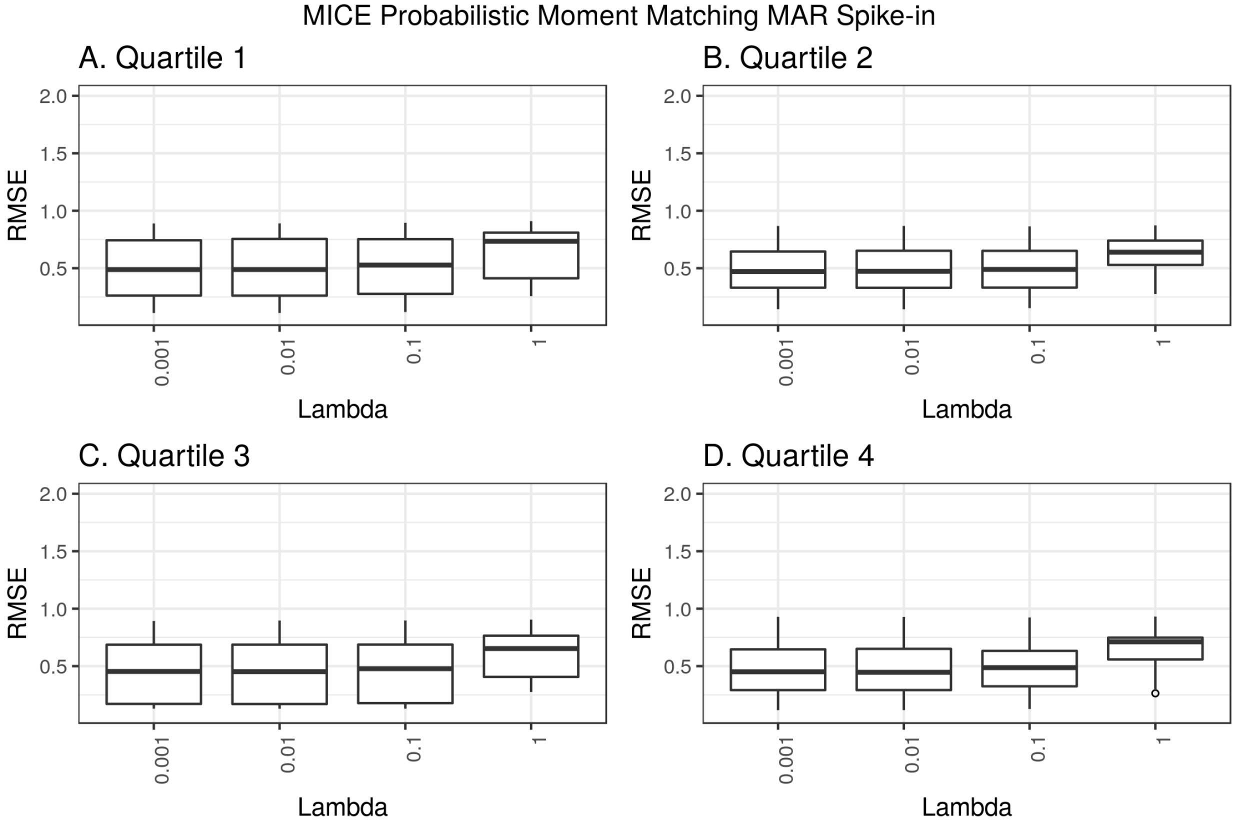

Supplemental Figure 20. MICE R package MAR Spike-in.

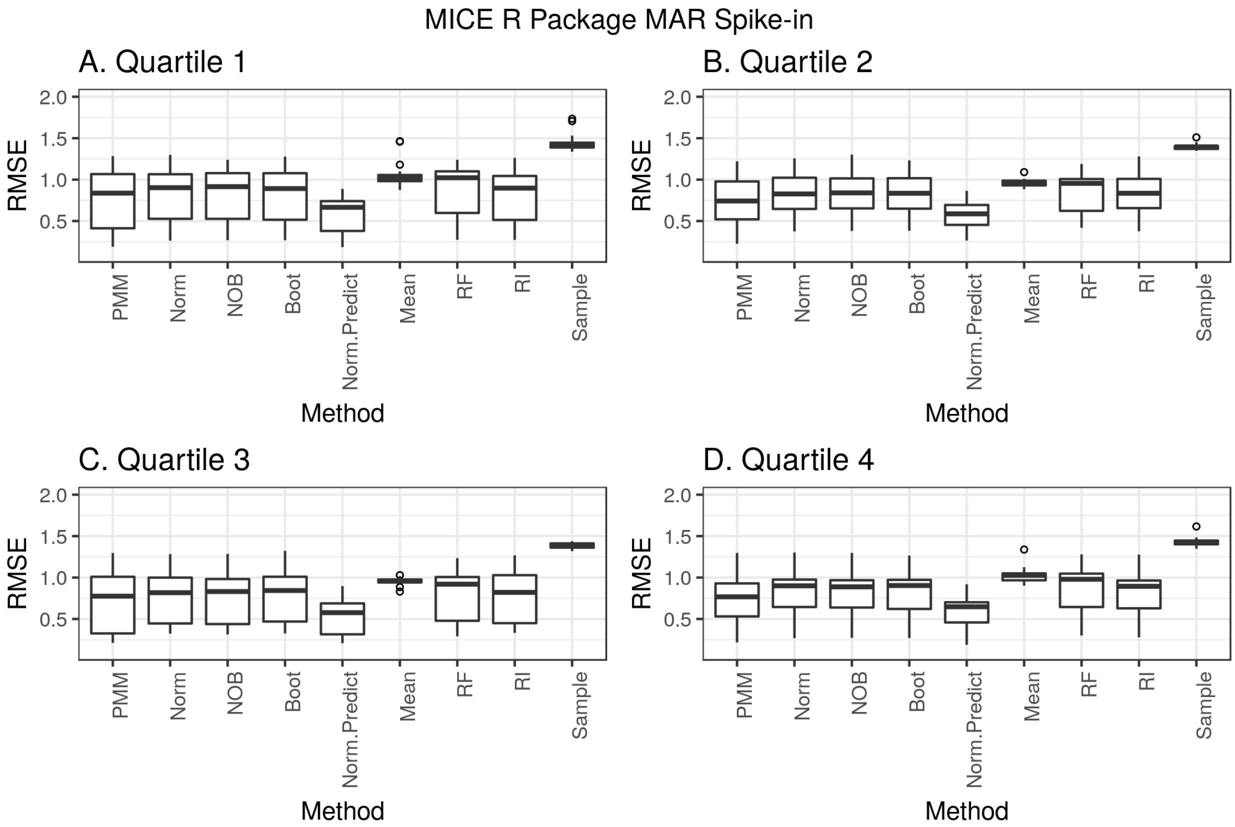

Supplemental Figure 21. Summary of imputation accuracy scores across all 10 sets of 10,000 patients.

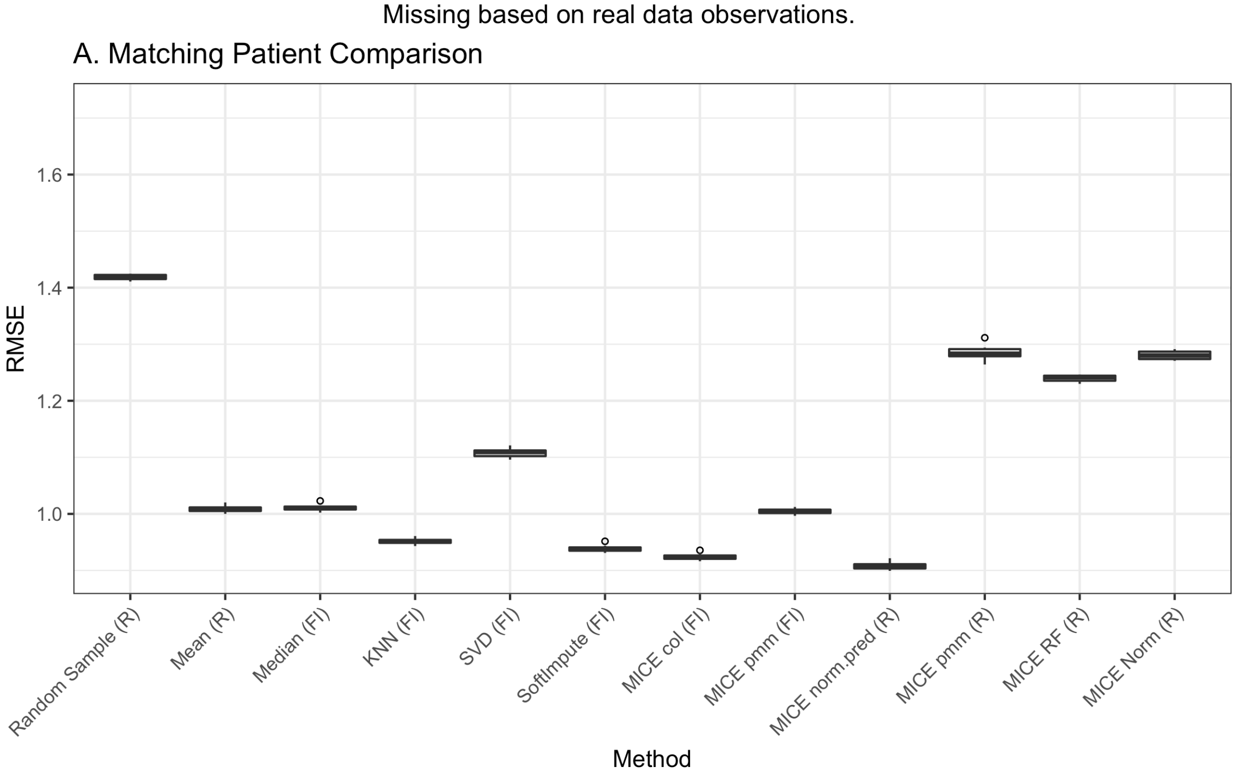

Supplemental Figure 22. Convergence of MICE PMM imputation chains

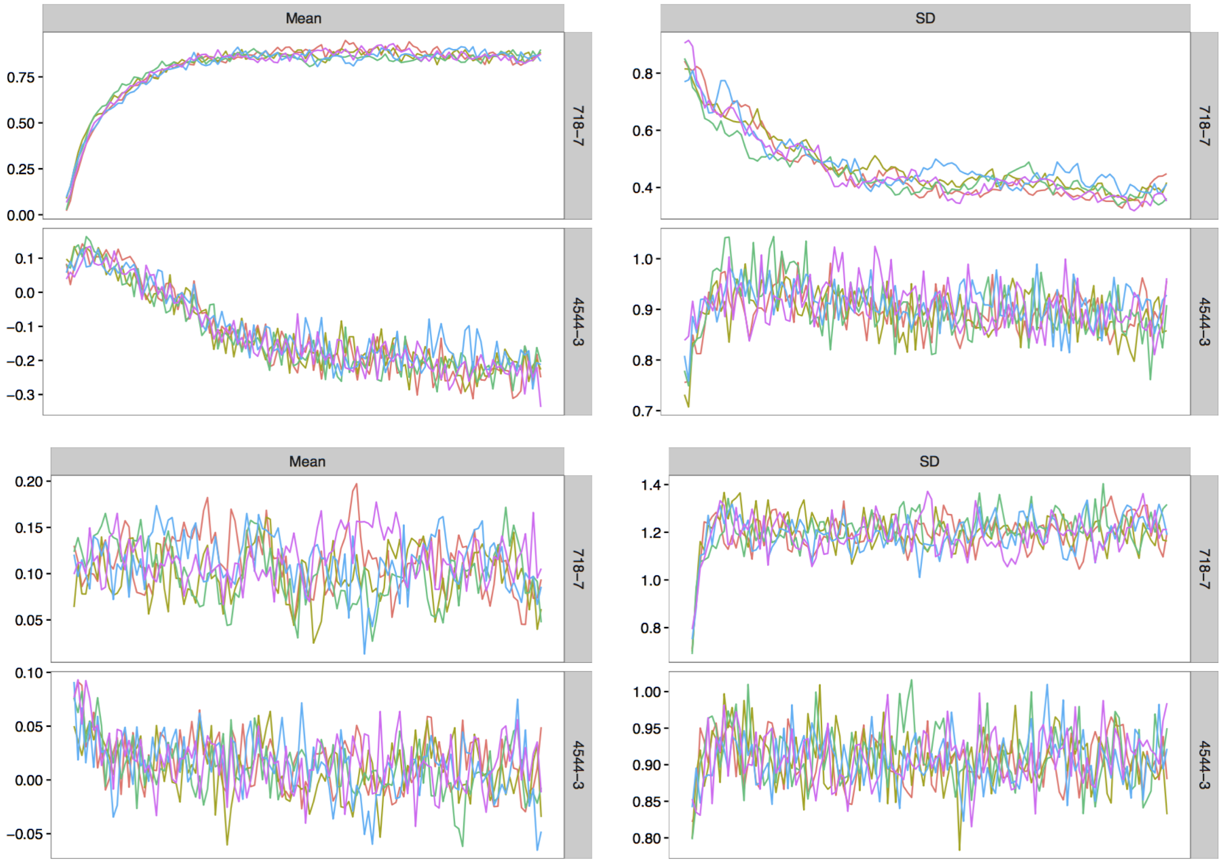

Assessment of convergence in the imputed values using the pmm method in the mice package. Five imputations were run for 125 iterations. The mean and standard deviations of the imputed values were plotted as a function of the iteration number. This was performed for each variable but two examples are shown above. The top for panels show a failure of convergence when all variables were included in the predictor matrix. The bottom four panels show convergence within 20 iterations when highly correlated variables were not used simultaneously as predictors. The means and standard deviations of the imputation chains show no trends after 20 iterations and the individual chains are indistinguishable.
